# Copper resistance in the cold: Genome analysis and characterisation of a P_IB‐1_ ATPase in *Bizionia argentinensis*

**DOI:** 10.1111/1758-2229.13278

**Published:** 2024-06-28

**Authors:** Noelia I. Burgardt, Noelia A. Melian, F. Luis González Flecha

**Affiliations:** ^1^ Laboratorio de Biofísica Molecular, Facultad de Farmacia y Bioquímica, Instituto de Química y Fisicoquímica Biológicas Universidad de Buenos Aires, Consejo Nacional de Investigaciones Científicas y Técnicas Buenos Aires Argentina; ^2^ Present address: Departamento de Ciencia y Tecnología Universidad Nacional de Quilmes Bernal Argentina

## Abstract

Copper homeostasis is a fundamental process in organisms, characterised by unique pathways that have evolved to meet specific needs while preserving core resistance mechanisms. While these systems are well‐documented in model bacteria, information on copper resistance in species adapted to cold environments is scarce. This study investigates the potential genes related to copper homeostasis in the genome of *Bizionia argentinensis* (JUB59‐T), a psychrotolerant bacterium isolated from Antarctic seawater. We identified several genes encoding proteins analogous to those crucial for copper homeostasis, including three sequences of copper‐transport P1B‐type ATPases. One of these, referred to as BaCopA1, was chosen for cloning and expression in *Saccharomyces cerevisiae*. BaCopA1 was successfully integrated into yeast membranes and subsequently extracted with detergent. The purified BaCopA1 demonstrated the ability to catalyse ATP hydrolysis at low temperatures. Structural models of various BaCopA1 conformations were generated and compared with mesophilic and thermophilic homologous structures. The significant conservation of critical residues and structural similarity among these proteins suggest a shared reaction mechanism for copper transport. This study is the first to report a psychrotolerant P1B‐ATPase that has been expressed and purified in a functional form.

## Graphical abstract

This study explores copper homeostasis in *Bizionia argentinensis*, a psychrotolerant bacterium from Antarctic seawater. The genome reveals genes similar to known copper homeostasis proteins, including a P1B‐type ATPase, BaCopA1. Expressed in yeast, BaCopA1 catalyses ATP hydrolysis at low temperatures. Its structural similarity to other proteins suggests a shared copper transport mechanism.

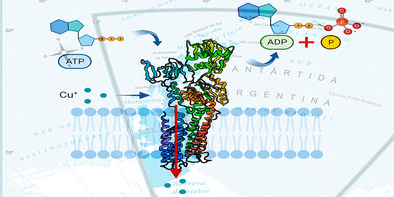

## INTRODUCTION

The role of copper ions in biological processes spread out when atmospheric molecular oxygen increased because insoluble Cu(I) was oxidised to the more soluble and bioavailable Cu(II) (Festa & Thiele, 2011). Both copper ions are found as cofactors and prosthetic groups of numerous enzymes and bind to transport‐ and storage proteins. Because high levels of intracellular copper ions can induce DNA, protein and lipid damage, specific biological mechanisms have evolved to regulate copper ions uptake, mobilisation, storage and elimination (Boal & Rosenzweig, 2009; Maung et al., 2021). Many protein families associated with copper homeostasis—such as influx and efflux transmembrane (TM) transporters, metallochaperones and metalloregulatory proteins—are found in all domains of life.

Bacterial copper homeostasis is focused on protection against free‐radical damage, with functions related to sensing, intracellular mobilisation and elimination. Gram‐negative bacteria transport copper ions through the outer membrane into the periplasm by porins and/or TonB‐dependent proteins. The copper ions periplasmic pool is managed by chaperones, storage proteins and chemical chelators (Andrei et al., 2020). Periplasmic metal overload response is controlled by a conserved two‐component system, which comprises a membrane sensor histidine‐kinase and a cytoplasmic response regulator partner (Novoa‐Aponte et al., 2020). The uptake of copper ions across the plasmatic membrane is achieved by Major‐Facilitator Superfamily members, using the proton gradient as the driving force. Once inside the cell, proteins bind copper ions with high affinities and transfer these ions via ligand exchange, warranting that copper ions are always complexed. The excess of the intracellular copper ions is eliminated from the cytosol by active transporters such as P‐type ATPases (Inesi, 2017). Copper elimination is controlled by transcriptional and post‐transcriptional regulation, allosteric modulation and targeted proteolysis. Regulation of copper levels in bacterial cells is achieved mostly by transcriptional control by modification of the transporter abundance (Argüello et al., 2013).

Copper resistance mechanisms in bacteria are focused on Cu(I) elimination, with the P_IB‐1_‐ATPases a key player in this process. These pumps transport the metal ion across the cell membrane using ATP hydrolysis as a driving force, with the formation of a phosphorylated intermediate during the reaction cycle. The general topology of these polytopic membrane proteins includes a large TM domain and three or more cytoplasmic domains (Recoulat Angelini et al., 2021). The TM domain has eight α‐helices arranged in a helix bundle, establishing the ion's pathway with a TM metal‐binding site (residues CPC). The cytoplasmic domains are the catalytic domain, the actuator domain and the heavy metal‐associated (HMA) domains. The catalytic domain is formed by two subdomains: the nucleotide‐binding domain and the phosphorylation domain, which encloses the highly conserved phosphorylation site (residues DKTG). The actuator domain participates in the catalytic cycle through specific interactions with the catalytic domain. One or more soluble metal‐binding domains may be present, usually at the amino‐terminal end, which in most cases have a highly conserved metal binding motif (residues CXXC) (Palmgren, 2023). The general enzymatic mechanism of P_IB‐1_‐ATPases can be described by the Albers‐Post model, which considers the existence of two enzyme conformations denoted as E1 and E2 (Recoulat Angelini et al., 2021 and references therein). Briefly, the cycle starts when the inward‐facing E1 conformation binds Cu(I) and ATP (Figure S1), giving the E1‐ATP form. After nucleotide hydrolysis the enzyme gets phosphorylated and the E1P conformation is obtained. Then, the outward‐facing E2P state is formed, and the Cu(I) is released, leading to the dephosphorylated form (E2Pi). Finally, the phosphate anion is released and the pump returns to the E1 conformation closing the cycle. The structures of the Cu(I) transport ATPases from *Archaeoglobus fulgidus* (AfCopA; 7R0G, 7R0I) and *Legionella pneumophila* (LpCopA; 4BBJ, 3RFU) were identified as E1, E1‐ATP, E2P and E2Pi conformations (Andersson et al., 2014; Gourdon et al., 2011; Salustros et al., 2022) because their similarity to the structures of these conformations experimentally determined for the sarcoplasmic reticulum Ca^2+^‐ATPase (Sørensen et al., 2004). The functional characterisation of LpCopA was reported recently, showing a reaction model that shares the basic reaction steps of the Albers‐Post model, with two different binding affinities for a single ATP and the binding of at least two Cu(I) ions with different affinities (Placenti et al., 2022).

The mechanisms by which bacteria use and deliver copper metal ions show a wide biodiversity, but the main defence machinery is highly conserved. These systems are well described in model bacteria (Andrei et al., 2020), but there is little information on copper resistance in species adapted to cold environments. The Gram‐negative bacterium *Bizionia argentinensis* (JUB59‐T) was isolated from the surface marine water of Potter Cove, Antarctica (Bercovich et al., 2008), where copper ions concentrations were found to be high due to the glacier effluents (Andrade et al., 2001; Wiencke et al., 2008). This non‐motile aerobic bacillus grows within a temperature range of 2–28°C, with its optimal growth between 22 and 25°C, and no growth is observed above 30°C (Bercovich et al., 2008). *B. argentinensis* belongs to the family Flavobacteriaceae and shows high levels of sequence similarity with *Bizionia myxarmorum* ADA‐4T and *Bizionia algoritergicola* APA‐1T (Bercovich et al., 2008). The genome sequence for this psychrotolerant bacteria was reported, with 2.963 predicted open reading frames (ORFs) (Lanzarotti et al., 2011). This sequence analysis revealed the presence of genes for a complete tricarboxylic acid cycle, glycolysis, a pentose phosphate pathway and several genes related to the denitrification process. In this work, we report a description of the copper‐homeostasis gene repertoire in *B. argentinensis*, a bioinformatic analysis of the P_IB_‐ATPases sequences, and the cloning, expression and purification of a Cu(I) transport ATPase from this psychrotolerant bacterium.

## EXPERIMENTAL PROCEDURES

### *Analysis of* B. argentinensis *genome*

Proteins involved in bacterial copper resistance and/or homeostasis were identified from previous literature (Table S1) and then used as seed for protein–protein blast (Blast‐P) search restricted to *B. argentinensis* JUB59‐T genome to find similar sequences (Johnson et al., 2008). Also, Rapid Annotation using a Subsystem Technology server was used for blast search and preliminary analysis of genetic organisation (Andersson et al., 2014; Johnson et al., 2008). All the transcriptional unit (TU) were found with Operon Mapper (Taboada et al., 2018). The sequences were retrieved from the UniProt database (UniProt Consortium, 2023), the protein families and domains were identified using InterPro (Paysan‐Lafosse et al., 2023), multiple sequence alignment was performed with Clustal Omega (Sievers & Higgins, 2018), sequence identities and similarities were calculated using the LALIGN server (Huang & Miller, 1991). Conserved residues involved in the binding of Cu(I) ions were identified by sequence alignment. When necessary, signal peptides and their cleavage sites were predicted with the SignalP 6.0 server (Teufel et al., 2022), and the TM prediction was done with DeepTMHMM (Hallgren et al., 2022).

### Molecular cloning

The gene (WP_040288272, EGV43374.1, UniProt entry G2EDW3) coding the Cu(I) transport ATPase from *B. argentinensis* (BaCopA1) was amplified by PCR, using genomic DNA from *B. argentinensis* JUB59‐T as template (Lanzarotti et al., 2011) (accession number AFXZ 01000000) and the following primers:

Forward 5′TCGACGGATTCTAGAACTAGTGGATCCCCCATGACCTGCAACGGTTGTCGAAGTCACGTGGAA
Reverse 5′AAATTGACCTTGAAAATATAAATTTTCCCCTATTTTAATAGTTCTTAATCTTAAGGC.

Primers were designed soon after the release of the genomic sequence and the ORF for this protein was updated later, so the gene obtained with these primers represented the region from 10 to 840 of BaCopA1. The PCR product was incorporated into a plasmid that featured an inducible galactose (Gal) promoter, a Tobacco etch virus (TEV) cleavable His6 fusion tag at its C‐terminus, and a green fluorescent protein (GFP) as a marker for gene expression. This vector, denoted as p424Gal‐TEVGFPhis8, was introduced into *S. cerevisiae* FGY217 cells through the mechanism of homologous recombination (Oldenburg et al., 1997). Briefly, the vector was digested with SmaI for linearisation and then transformed into the yeast cells together with the PCR product using the lithium acetate method (Gietz & Woods, 2002). The transformed cells were grown in a URA plate (0.67% YNB, 0.08% CSM‐URA, 2% glucose, 2% agar) at 30°C and single colonies were selected (Drew et al., 2008). The final construct was isolated from yeast cells using a modified miniprep protocol and sequenced.

### Protein expression and purification

Yeast culture was grown at 28°C, in medium YEP‐0.1% glucose (1% back‐tryptone, 1% yeast extract) or ‐URA medium (0.17% YNB, 0.08% CSM‐URA, 0.5% ammonium sulfate, 2% glucose, 2% agar). Protein expression was induced by the addition of 2% Gal when the optical density at 600 nm reached 0.6 units (Drew et al., 2008). Whole‐cell GFP‐Fluorescence was registered using a JASCO FP‐6500 fluorometer and used as an indicator of the level of protein expression. The yeast culture was harvested after 24 h of induction (by sedimentation at 2000 g for 10 min using a Sorvall RC5B centrifuge) and the cells were disrupted with bead glasses in a medium containing 1× YSB (50 mM Tris–HCl, 10% Glycerol, pH 7.5), 1 mM EDTA, 2 mM ß mercaptoethanol and 1 mM phenylmethylsulfonyl fluoride. The membrane fraction was isolated by sedimentation at 23,000 g for 60 min using a Sorvall RC5B centrifuge and then solubilised in the same medium used for cell disruption with 0.5% *n*‐dodecyl‐beta‐maltoside. BaCopA1 was purified by affinity chromatography using a Ni^2+^‐nitrilotriacetic acid column (Ni‐NTA, BioRad) equilibrated with YSB and decaethylene glycol monododecyl ether (C_12_E_10_). After purification, the protein was concentrated to 0.1–0.5 mg/mL using a vivaspin concentrator with 50 kDa cut off (Sartorius) and stored in 100 mM Tris–HCl (pH 7.5 at 25°C), 20% glycerol, 100 mM NaCl, 0.05% C_12_E_10_ and stored at −80°C until use. After purifying BaCopA1, we did not remove both GFP and the His‐tag for subsequent biochemical analyses. Due to the limited protein yield, attempts to remove GFP were unsuccessful. Previous biochemical studies on other P‐ATPases suggest that attaching fluorescent proteins such as GFP at either the N‐terminus or the C‐terminus does not significantly affect ATPase activity compared to the unfused protein (Corradi et al., 2016; Winters et al., 2008). Protein integrity and purity were determined by Tris‐Tricine SDS–PAGE (Schägger, 2006). *S. cerevisiae* membranes or purified protein samples were incubated at room temperature in Sample Buffer (80 Tris mM pH 6.8, 2% SDS, 10% glycerol, 0.0006% bromophenol blue) and loaded in the gel without boiling. In‐gel GFP–Fluorescence was determined by blue and UV epi‐illumination (ImageQuant LAS500, General Electric) of the unstained gels. In the fluorescence image, the molecular weight markers are visible because they were blue‐prestained (Bio‐Rad). Afterwards, the Coomassie Brilliant Blue stain was performed (Neuhoff et al., 1988). The concentration of each protein preparation was measured by SDS–PAGE densitometry, using ImageJ 1.53 t and BSA as standard (Alonso Villela et al., 2020).

### Activity assays

ATPase activity was measured as the initial rate of release of inorganic phosphate (Pi) from ATP at 5°C (or the temperatures indicated in Figure 4) in a medium containing: 50 mM Tris–HCl, pH 8.0 at the working temperature, 2.0 mM MgCl_2_, 2.0 mM ATP, 0.1 mM CuSO_4_, 20 mM cysteine, 0.02% asolectin, 0.096% C_12_E_10_, 50 mM NaCl, 2 mM azide and 11 μg purified enzyme. Specific modifications in the reaction medium composition are indicated in the legend of Figure 5. Released Pi was quantified using a malachite green procedure (Martínez Gache et al., 2020; Recoulat Angelini et al., 2022).

### Molecular modelling

BaCopA1 homology models were generated using Phyre2 (Kelley et al., 2015), MODELLER (Webb & Sali, 2016), tr‐Rosetta (Baek et al., 2021; Du et al., 2021), AlphaFold 2 (Jumper et al., 2021) and RosettaFold (Baek et al., 2021; Mirdita et al., 2022). In all cases, the amino acid sequence of BaCopA1 without the N‐terminal (residues from 174 to 840) was used as input because this domain was not solved in the structures of LpCopA and AfCopA. The structure of LpCopA (PDB ID 3RFU, chain A) was chosen as the template in Phyre2. The structures obtained with Phyre2 and MODELLER were refined with ModRefiner (Xu & Zhang, 2011). The initial models obtained with Tr‐Rosetta and Alpha Fold 2 have a feature unlikely to be accurate, i.e. the formation of a disulfide bridge between the cysteines of the TM copper‐binding site (Cys483‐Cys485). Then, these models were first modified to break these disulfide bridges and refined later. Evaluation of structure quality was achieved for each model with MolProbity (Williams et al., 2018). The structural similarity of BaCopA1 models with the experimental structures was determined by comparison of root mean square deviation values obtained from the structural alignment using Pymol viewer (https://pymol.org/2).

## RESULTS AND DISCUSSION

### *Copper homeostasis in* B. argentinensis *(JUB59‐T)*

**FIGURE 1:**
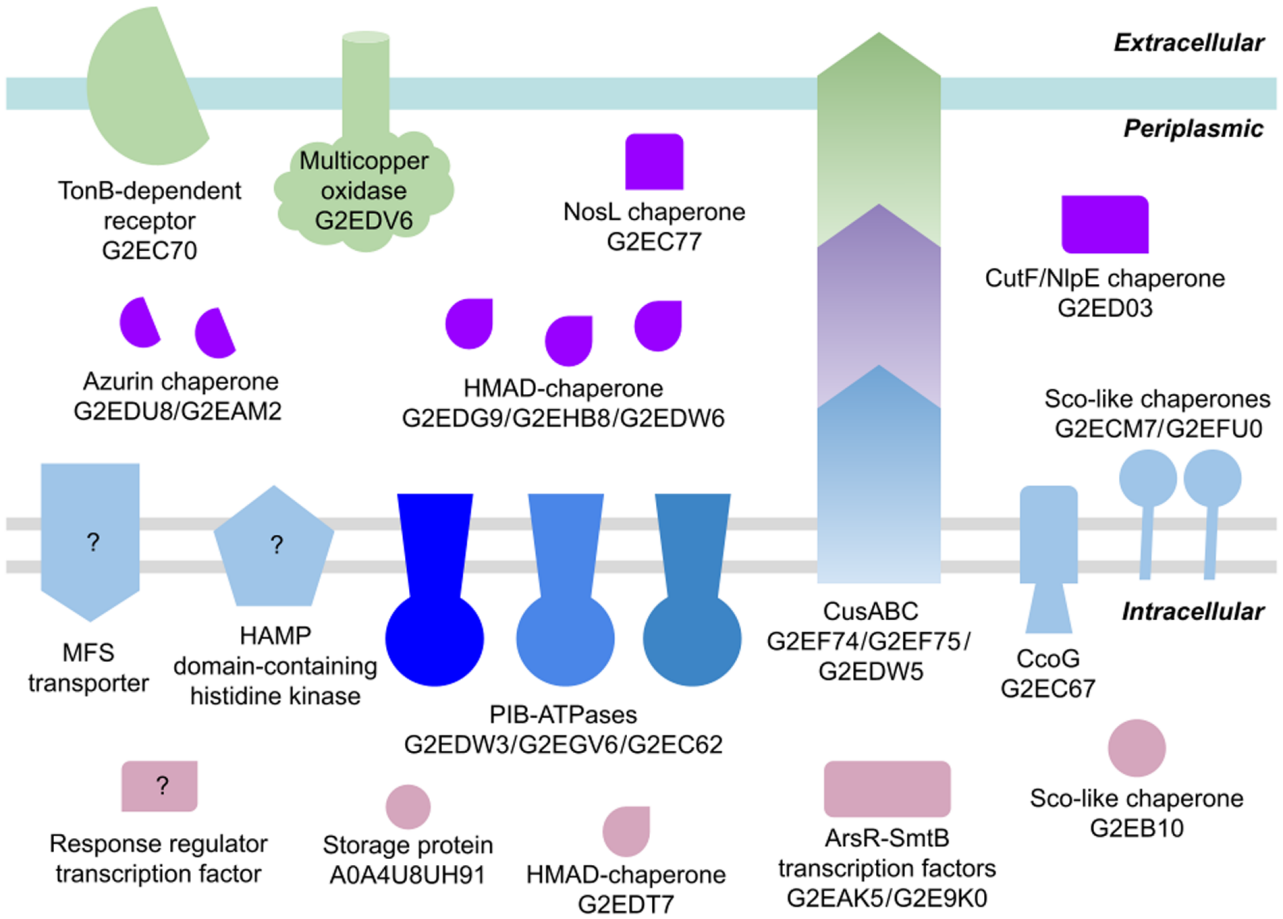
Schematic representation of proteins found in *Bizionia argentinensis* genome and their hypothetical function in copper homeostasis. Several proteins possibly associated with the transport, transfer, uptake and/or storage of copper ions have been identified across all cellular compartments. The name and UniProt code for each protein is indicated. Proteins integrated into the outer membrane (green shades) might play a role in the copper ions influx, efflux and redox processes. Within the periplasm, ions might be captured and stored by a variety of soluble chaperones (purple shade). Also, the periplasmic subunit of an RND transporter might be associated with ions efflux (purple). Proteins embedded in the inner membrane (blue and light‐blue colour shades) might participate in the influx and efflux of copper ions into the cytoplasm, in the regulation responses, and redox reactions. Finally, a set of cytoplasmic proteins (pink shades) might perform essential functions in response regulation, uptake, storage and/or transfer, creating a continuous flux of ions along different pathways based on cellular requirements.

The marine environment from where *B. argentinensis* was isolated contains a high concentration of copper ions due to the erosion of volcanic rocks nearby natural glacier (Andrade et al., 2001; Wiencke et al., 2008). Hereby, the examination of its genome sequence shows a wide repertoire of genes that might be related to copper homeostasis (Figure 1, Table 1). These genes were found performing BLAST searches restricted to the *B. argentinensis* genome, using as seed different proteins known to be involved in copper homeostasis in other Gram‐negative bacteria (Table S1).

**TABLE 1:** *Bizionia argentinensis* proteins that are possibly involved in copper homeostasis.

TU	Gene	UniProt	Description	Hypothetical function
124	BZARG_211	G2E9K0	ArsR family transcriptional regulator	Cytoplasm regulationG2EC67
326	BZARG_2939	G2EAK5	ArsR family transcriptional regulator	Cytoplasm regulation
336	BZARG_2956	G2EAM2	Azurin	Periplasm resistance
**618**	**BZARG_1486**	**G2EC62**	**P‐type ATPase**	**Elimination**
619	BZARG_1491	G2EC67	CcoG	Intracellular resistance
620	BZARG_1494	G2EC70	TonB‐dependent receptor	Uptake
623	BZARG_1501	G2EC77	Copper chaperone NosL	Periplasm biogenesis
704	BZARG_2979	G2ECM7	SCO family protein	Periplasm biogenesis
780	BZARG_1273	G2ED03	META‐D‐containing protein	Periplasm biogenesis
887	BZARG_1319	G2EDG9	HMAD‐containing protein	Copper chaperone
940	BZARG_1753	G2EDT7	HMAD‐containing protein	Copper chaperone
946	BZARG_1764	G2EDU8	Azurin	Periplasm resistance
950	BZARG_1769	G2EDV3	RND transporter subunit	Elimination
951	BZARG_1772	G2EDV6	Multicopper oxidase	Periplasm resistance
**953**	**BZARG_1779**	**G2EDW3**	**P‐type ATPase**	**Elimination**
953	BZARG_1781	G2EDW5	RND transporter subunit	Elimination
953	BZARG_1782	G2EDW6	HMAD‐containing protein	Copper chaperone
1212	BZARG_2009	G2EF75	RND transporter subunit	Elimination
1319	BZARG_1951	G2EFU0	SCO family protein	Periplasm biogenesis
**1522**	**BZARG_2573**	**G2EGV6**	**P‐type ATPase**	**Elimination**
**1604**	**BZARG_491**	**G2EHA4**	**P‐type ATPase**	**Elimination**
1611	BZARG_505	G2EHB8	HMAD‐containing protein	Copper chaperone
*	BZARG_03225	A0A4U8UH91	Four‐helix bundle copper‐binding protein	Intracellular resistance

*Note*: Proteins are arranged in the table by their genome location, showing their transcriptional unit number (TU), gene names (locus tag NCBI), UniProt codes and a brief description of the hypothetical function derived from bioinformatics studies. Genes coding for P‐ATPases are highlighted in bold.

Abbreviation: HMAD, heavy metal‐associated domain.

Copper uptake across the outer membrane in *B. argentinensis* might be performed by a TonB‐dependent Transporter (UniProt G2EC70), which has 21.8% identity and 53.1% similarity to OprC from *Pseudomonas aeruginosa* (UniProt P72121). Also, the conserved metal‐binding motif found in OprC (CxxxM‐HxM) is present in this protein (Bhamidimarri et al., 2021). There are other TonB‐dependent proteins in the genome, but they do not have similarity to OprC and do not encode the metal binding motif.

The genome of *B. argentinensis* lacks proteins associated with copper ions incorporation through the outer membrane, such as porins (e.g. *Escherichiacoli* OmpF/OmpC) or proteins associated with indirect copper ion transport (e.g. *Methylococcus capsulatus* MbnT). The absence of these genes may represent an adaptation related to copper resistance, possibly aimed at minimising the uptake of these ions. Notably, studies on *E. coli* mutants lacking OmpF have demonstrated copper resistance (Lutkenhaus, 1977; Maung et al., 2021).

Periplasm regulation of copper concentration ions in *B. argentinensis* seems to be achieved by different mechanisms. The putative periplasm Multicopper Oxidase (G2EDV6) might play a crucial role in copper detoxification (Grass & Rensing, 2001). Its sequence shows 62.1% similar and 35.7% identity with *E. coli* copper resistance protein A (PcoA, Q47452) and 62.3% similar and 34.2% identity with copper resistance protein A (P12374) from *Pseudomonas syringae pv. tomato*. Copper ion entry and copper binding sites are conserved with the *E. coli* PcoA and CueO homologues (Table S1, Figure S2) (Roberts et al., 2002). Residues 1–19 are predicted to be a signal peptide for periplasm localisation, the region from 200 to 610 contains three Plastocyanin‐like domains (PF00394: Cu‐oxidase 1, PF07731:Cu‐oxidase 2 1 and PF07732: Cu‐oxidase 3) and the C‐terminal residues 611–809 seem to be a porin‐like region (Porins homologous superfamily, InterPro SSF56935) with some similarity to PcoB from *E. coli*.

Two copper chaperones of *B. argentinensis* (G2EDU8 and G2EAM2) might act in the periplasm storage and transport of these ions. They are similar to the periplasmic plastocyanin‐like CopI from *Rubrivivax gelatinosus* (W8FLH9), and they both have a periplasmic signal‐peptide and the putative copper‐binding motif present in CopI (Durand et al., 2015). The search for other periplasmic copper storage proteins similar to *Neisseria gonorrhoeae* Csp1 (Q5F665) and the periplasmic copper chaperones similar to *Salmonella typhimurium* CueP (Q8ZL99) and *E. coli* CusF (P77214) was negative.

Some periplasmic chaperones for cuproprotein biogenesis related to the Sco family (G2ECM7 and G2EB10), NosL (G2EC77) and CutF (G2ED03) were found in *B. argentinensis* and maintain the conserved copper‐binding site reported for each protein (Ekim Kocabey et al., 2021; Öztürk et al., 2021, Prasser et al., 2021) (Figure S3). The putative Sco‐like chaperones G2ECM7 and G2EFU0 are similar to the chaperones SenC from *Rhodobacter capsulatus* (Q52720) and *Bacillus subtilis* (P54178). These proteins have one amino‐terminal TM helix, suggesting that they are anchored to the membrane and they share the conserved copper‐binding motif CxxxC characteristic of this family (Balatri et al., 2003). Besides their putative role as assembly factors for the biogenesis of Cbb3‐type Cytochrome Oxidase, they might be involved in oxidative stress response. There is another protein of *B. argentinensis* with a Sco‐like domain and an N‐terminal heavy‐metal binding domain (G2EB10) that does not have a signal peptide, so it might be intracellular. Putative NosL (G2EC77) is similar to *P. aeruginosa* NosL protein (Q9HYK8) and to *Shewanella denitrificans* NosL (Q12M31). It has a lipoprotein signal peptide (Sec/SPII) for periplasm localisation, and most of the residues identified as the metal‐binding site in *S. denitrificans* NosL are conserved (Prasser et al., 2021). CutF/NlpE found in *B. argentinensis* (G2ED03) is similar to *E. coli* CutF (A7ZHT3) and has the putative copper‐binding motif CxxC (Simpson & Trent, 2019). Periplasm regulation of copper ion levels in *B. argentinensis* might be monitored by two‐component systems similar to CusRS or CopRS. Three putative proteins (G2EHH2, G2EGQ3 and G2EBH0) have similarities to sensor histidine kinases like CusS from *E. coli* (P77485) and CopS from *P. aeruginosa* (Q02541). These putative sensor histidine kinases have high sequence similarity only with the cytoplasmic histidine kinase domains of CusS and CopS, but they do not share similarities in the periplasm domains where the metal‐binding site is located. The protein G2EHH2 does not have a periplasmic domain and the proteins G2EGQ3 and G2EBH0 have periplasmic domains with no homology to any known domain. There are three putative proteins (G2EGV3, G2EGQ4 and G2EHH1) similar to copper transcriptional regulatory proteins such as CusR from *E. coli* (P0ACZ8) and CopR from *P. aeruginosa* (Q9I034). They all have an N‐terminal response regulatory domain followed by an OmpR/PhoB‐type DNA‐binding domain and belong to the transcriptional regulatory protein *WalR‐like* Protein Family (IPR039420). Although G2EGV3 shows the highest identity with CusR and CopR, it may not be related to copper regulation because it shares the TU with a sensor kinase of unknown function (G2EGU9). Also, G2EHH1 does not seem to be involved with copper sensing because it is located in the same TU as G2EHH2, the sensor histidine kinase that does not have a periplasmic sensor domain. Then, G2EGQ4 might have a role in copper regulation matching with the sensor histidine kinases G2EGQ3.

The uptake of copper ions through the plasma membrane of *B. argentinensis* remains uncertain, none of the sequences identified in the genome as Major‐Facilitator Superfamily proteins have the copper binding motif conserved in CcoA copper transporters. Some of these have several methionines and histidine residues in the TM helices 7 and 8, where the copper‐binding site of CcoA is located, so they could constitute an atypical Cu(I) transporter.

Once inside the cytoplasm, copper ions can be stored and/or delivered by intracellular storage proteins and chaperones. Four proteins containing a highly conserved copper‐binding site (Utz et al., 2019) associated with a heavy metal domain were identified in the *B. argentinensis* genome, all displaying similarity to the bacterial copper chaperone (CopZ) family. Three of them might be located at the periplasm because they have a signal peptide (G2EDW6:BaCopZP1, G2EDG9:BaCopZP2, G2EHB8:BaCopZP3) and have similarities with MerP proteins from *Pseudomonas* sp. K‐62 (O07301), *Pseudomonas stutzeri* (O66016), and *Serratia marcescens* (P13113). Therefore, only one of these putative CopZ might be present in the cytoplasm of *B. argentinensis* (G2EDT7: BaCopZ).

A membrane‐bound protein similar to CcoG from *R. capsulatus* (O30731) is present in the *B. argentinensis* genome (G2EC67). This protein could be involved in Cu(II) reduction, providing Cu(I) for cbb3‐Cox assembly, Cu‐chaperones and P_IB_‐type ATPases (Marckmann et al., 2019).

A copper‐storage intracellular protein similar to YhjQ from *B. subtilis* (O07571) was found in *B. argentinensis* (A0A4U8UH91). The structure of YhjQ (5FIG) shows a four‐helix bundle and a conserved copper‐binding motif (Vita et al., 2016).

Some transcriptional factors might be involved in the regulation of copper cytoplasm levels in *B. argentinensis*. There are two MerR transcriptional regulators (G2EA47, G2E8Y3); they both have an HTH merR‐type domain in the N‐terminal region, but they do not have the copper‐binding site reported for *E. coli* CueR (Changela et al., 2003), so they may not be involved in the regulation of copper homeostasis. Indeed, recently, it was reported that one of these transcriptional regulators is involved in iron homeostasis (Pellizza et al., 2022). Three BlaI/MecI/CopY family transcriptional regulators with similarity to the transcriptional repressor CopY from *Enterococcus hirae* (Q47839) were found in the genome of *B. argentinensis* (G2EAK1, G2ED45 and G2EAI2), but none of them has conserved the CopY copper‐binding site. Additionally, two proteins belonging to the ArsR‐SmtB family of proteins (G2EAK5 and G2E9K0) were identified. They share sequence similarity with *Oscillatoria brevis* BxmR, a SmtB/ArsR family metalloregulator (Liu et al., 2004), and have two cysteines of the BxmR metal‐binding motif conserved.

Elimination of copper ions in *B. argentinensis* seems to be performed by RND transporters and P‐type heavy metal translocating ATPases. There are 13 proteins identified as RND subunits. Four efflux RND transporters (G2EHA6, G2EDV3, G2EF74 and G2EHL7) are similar to CusA from *E. coli* (P38054), but only one of these proteins (G2EDV3) has the residues required for metal ion transport (R83, E567, D617, E625, R669 and K678) and the three methionines (M573, M623 y M672) reported to be involved in copper ion binding in CusA (Long et al., 2010). The efflux RND transporter periplasmic adaptor subunit (G2EDW5) is similar to *E. coli* CusB (P77239), with a sequence identity of 23.3% and a similarity of 58.2%. Further analysis indicated that the protein has two of the three methionines involved in copper ion binding in CusB, instead of M64 it has a Cys. The efflux transporter outer membrane subunit (G2EF75) is similar to CusC (P77211), showing 23.8% sequence identity and 63.4% similarity.

*B. argentinensis* genome contains four genes with homology to P_IB_‐ATPases (Table 2), and all of them display the typical features of this family. They can be classified as P_IB‐1_ (G2EDW3, G2EGV6, G2EC62) and P_IB‐4_ types (G2EHA4) from the analysis of their conserved TM sequences (Figure S4) (Argüello, 2003). The P_IB1_‐ATPases from *B. argentinensis* are denominated BaCopA because this denomination is commonly used after their first discovery as protein products of the copper‐resistance operon (Cha & Cooksey, 1991; Rensing et al., 2000). The P_IB4_‐ATPase is named BaCzcP due to its similarity with CzcP from *Cupriavidus metallidurans* (Scherer & Nies, 2009).

**TABLE 2:** P_IB_‐ATPases identified in *Bizionia argentinensis*.

RefSeq	Gene	UniProt	Length	Type	Name
WP_040288272.1	BZARG_1779	G2EDW3	840	P_IB‐1_	BaCopA1
WP_008639365.1	BZARG_2573	G2EGV6	833	P_IB‐1_	BaCopA2
WP_008636154.1	BZARG_1486	G2EC62	790	P_IB‐1_	BaCopA3
WP_008639675.1	BZARG_491	G2EHA4	654	P_IB‐4_	BaCzcP

*Note*: NCBI reference sequence codes (RefSeq), gene names, UniProt codes and amino acid chain lengths are indicated. The P_IB_‐type classification was established through the identification of highly preserved motifs within transmembrane helices (Argüello, 2003). Among these genes, three are associated with Cu(I) transporters (P_IB‐1_ ATPases), and the fourth corresponds to a cobalt‐zinc‐cadmium‐associated pump (P_IB‐4_ ATPase).

Sequence comparison between these Cu(I) transport ATPases shows that BaCopA1 and BaCopA2 are the most similar (81.1% identity, 93.5% similarity), while BaCopA3 presents below 30% identity and nearly 60% similarity.

The genetic organisation of P_IB1_‐ATPases genes in *B. argentinensis* is not the same as the one described for other Gram‐negative bacteria (Figure S5), where the CopA gene is next to a copper chaperone and a transcriptional regulator (Bondarczuk & Piotrowska‐Seget, 2013). The genes BZARG_1779 (BaCopA1, G2EDW3) and BZARG_1782 (putative periplasmic CopZ, G2EDW6) are located in the TU 953, together with other genes for putative copper‐resistance proteins, like BZARG_1781 gene that codes for the CusB G2EDW5. Nearby, in the TU 940, can be found the gene BZARG_1753 that encoded the intracellular copper chaperone (CopZ, G2EDT7). The ORF BZARG_2573 (BaCopA2, G2EGV6) is alone in TU 1522, away from other genes related to copper resistance. BZARG_1486 (BaCopA3, G2EC62) is the only gene in TU 618, and BZARG_505 (putative periplasmic CopZ, G2EHB8) is located alone in TU 1611. These arrangements with several genes related to copper resistance nearby suggest that these P_IB‐1_‐ATPases might play a role in *B. argentinensis* metal resistance.

**FIGURE 2:**
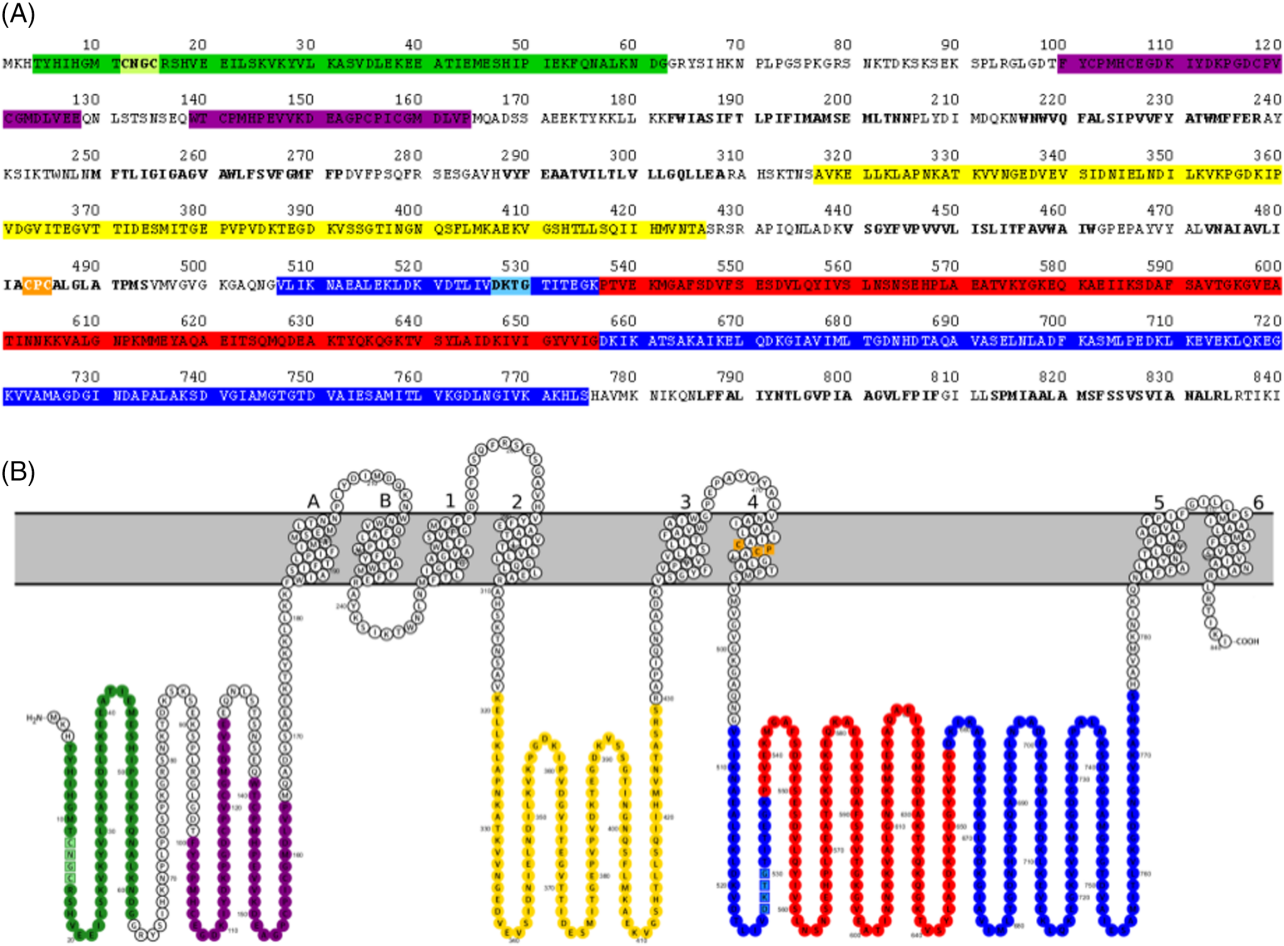
BaCopA1 (G2EDW3) amino acid sequence (A) and topology (B). The characteristic domains of P_IB‐1_‐type ATPases are highlighted: heavy metal associated domain (green), heavy metal binding domain (purple), actuator domain (yellow), phosphorylation domain (blue), nucleotide binding domain (red) and the eight predicted transmembrane helices (TM A‐B and 1–6). The conserved sequence motifs are also highlighted: metal binding site (CXXC, light green), transmembrane metal binding site (CPC, orange) and phosphorylation site (DKTG, light blue).

We selected BaCopA1 (G2EDW3) for cloning and protein expression because its sequence has high similarity to the well‐characterised Cu(I) transporters LpCopA (Q5ZWR1) and AfCopA (O29777) (Table S2). Furthermore, it is located in a TU with several putative copper‐related genes. BaCopA1 shares the typical topological pattern of P_IB‐1_ATPases: an N‐terminal heavy metal‐associated domain (residues 4–62), an actuator domain (residues 296–509), a catalytic domain which is composed of the phosphorylation domain and the nucleotide‐binding domain (ATPBD, residues 522–741) and a TM domain where it is located the Cu(I) TM binding motif (Figure 2). All the characteristic motifs of this type of ATPases (Argüello, 2003) are present in the BaCopA1 sequence, suggesting that this protein is involved in Cu(I) transport.

### Heterologous expression and purification of BaCopA1

Recombinant membrane protein expression and purification in the active form is not an easy job, even more so in the case of psychrotolerant proteins due to their intrinsic low stability (Carpenter et al., 2008; Feller, 2010). The traditional cloning method based on restriction enzyme molecular cloning (Sambrook & Russell, 2001) is difficult to monitor and time‐consuming, particularly in the case of membrane proteins. To avoid these problems, we choose to perform the cloning of BaCopA1 with a vector that fuses GFP at the C‐terminal by homologous recombination in *S. cerevisiae*, well described by Drew and collaborators (Drew et al., 2008). This approach allows the evaluation of protein expression and localisation through GFP fluorescence, either in yeast cultures or even in gel after SDS–PAGE (Drew et al., 2008). After cloning, GFP fluorescence was detected in the yeast membrane fraction (Figure S6). In‐gel GFP fluorescence showed a band corresponding to BaCopA1 molecular weight, indicating that this protein was successfully located in the membrane fraction (Figure S6).

**FIGURE 3:**
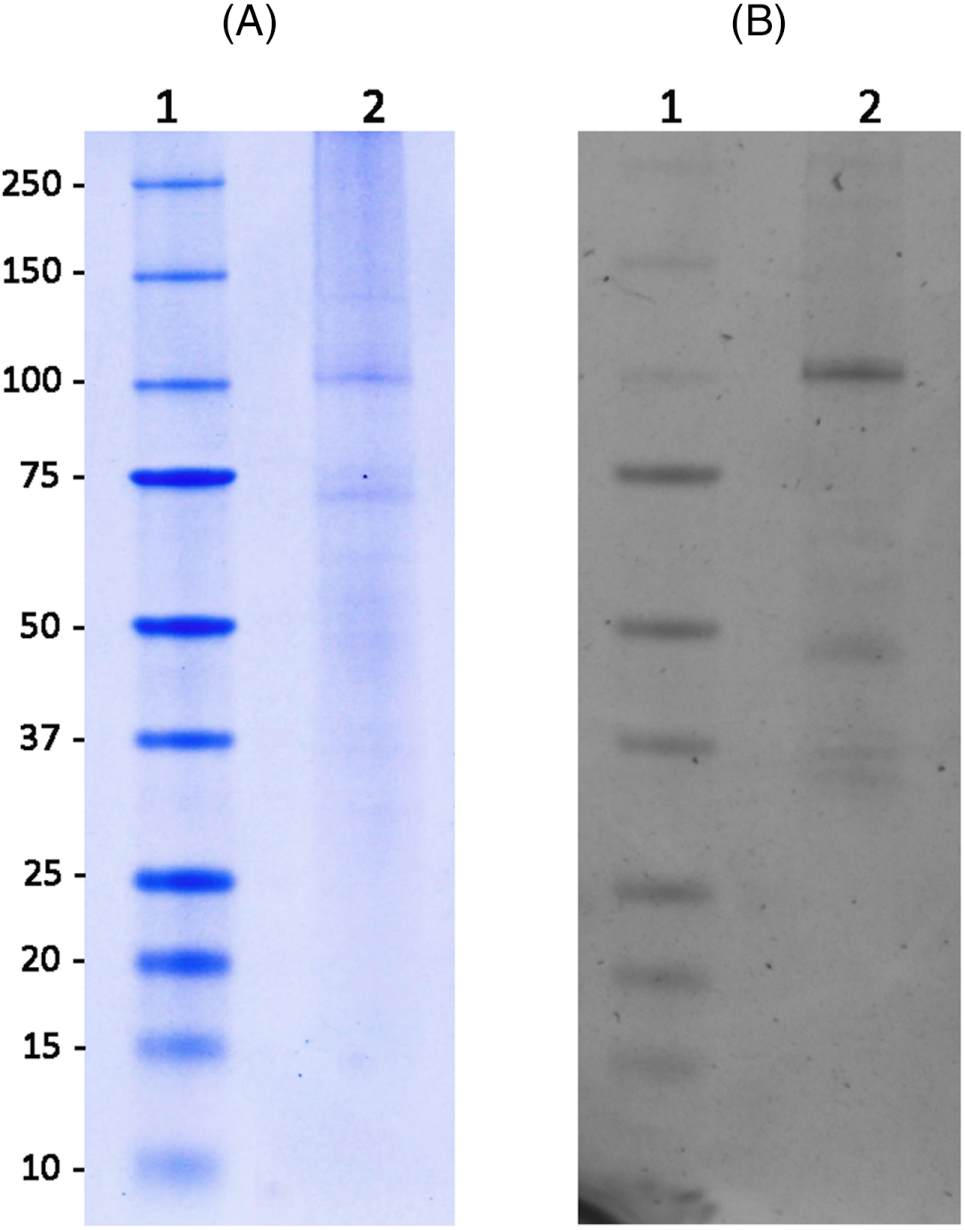
Purified BaCopA1 purity and integrity. SDS–PAGE analysis was performed as described in Experimental Procedures. Coomassie blue stained gel (A) and UV–vis epi‐illuminated unstained gel (B) are shown. The molecular weight prestained markers are in lane 1, with the corresponding molecular weights indicated at the side and the BaCopA pool of fractions from Ni‐NTA purification is in lane 2. BaCopA1‐GFP is the band above 100 kDa, visible in both panels. Due to low protein expression levels, the purified BaCopA1 sample shows some impurities, low concentration and the presence of degradation products, which are detected by GFP‐fluorescence.

A detergent screening assay for membrane protein solubilisation indicated that *n*‐dodecyl‐beta‐maltoside was the most effective. It was able to solubilise almost 80% of BaCopA1 from the membranes as observed by GFP fluorescence measurements (Figure S6). After solubilisation, Ni‐NTA chromatography using C_12_E_10_ as detergent was performed and the result was analysed by GFP fluorescence (Figure 3).

### Cu(I)‐ATPase activity of BaCopA1

**FIGURE 4:**
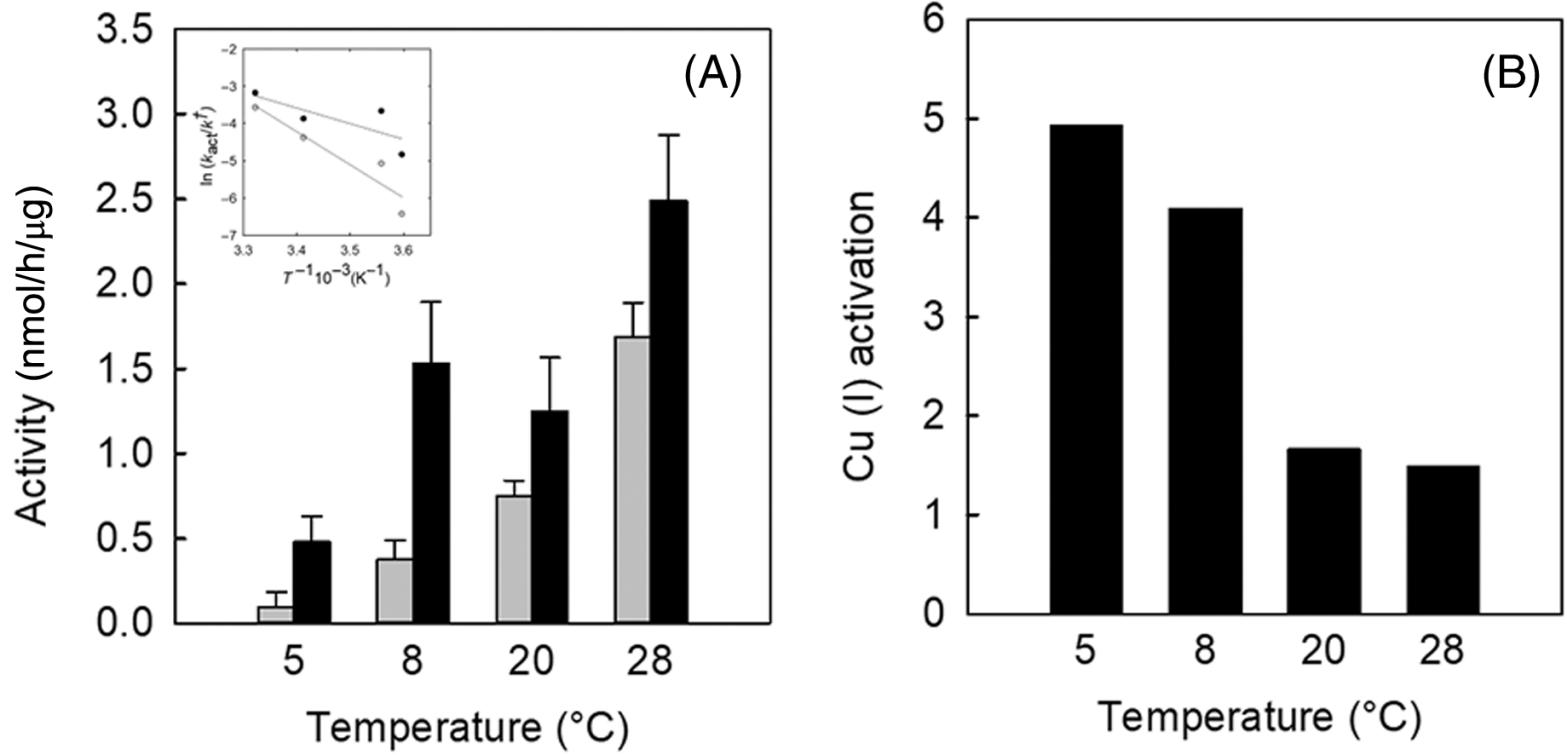
ATPase activity of BaCopA1 at different temperatures. (A) The ATPase activity in the absence (grey bar) and presence of Cu(I) (black bar) at different temperatures (5, 8, 20 and 28°C). The procedure for activity assays is described in the Experimental Procedures section. Error bars are the standard error of ATPase activity calculations. The inset in Panel (A) shows the corresponding Arrhenius plot for ATPase activity values with and without Cu(I), with the same colour reference. The solid line represents the fit of the linearised Arrhenius equation for each condition. (B) Cu(I) activation for each temperature condition. This activation factor is calculated as the ratio between ATPase activity in the presence and absence of Cu(I).

Purified BaCopA1 can catalyse ATP hydrolysis at low and moderate temperatures (Figure 4A). ATPase activity was measured as the initial rate of ATP hydrolysis at several temperatures using the reaction medium described in Experimental Procedures in the presence and absence of Cu(I). Because membrane enzymes irreversibly inactivate at all incubation temperatures (González Flecha, 2017), it is critical to use only the linear portion of the time courses of the release of Pi for the activity assays.

It can be observed that, although ATP hydrolysis increases with temperature in the range shown in Figure 4A, Cu(I) activation decreases (Figure 4B). Then, to further characterise the Cu(I)‐ATPase activity, the temperature of 5°C was selected.

Two controls were performed before analysing the modulation of the ATPase activity of BaCopA1. In the first one, we measured the time course of ATP hydrolysis in the same reaction medium without the enzyme and found that the rate of non‐enzymatic hydrolysis is at least one order the magnitude lower than that corresponding to the enzyme‐catalysed hydrolysis. In the second control, the ATPase activity was measured in the presence of CuSO_4_ but without the addition of the reducing‐complexing agent cysteine, and it was found that the rate of hydrolysis was the same as that observed in the absence of the copper ions.

**FIGURE 5:**
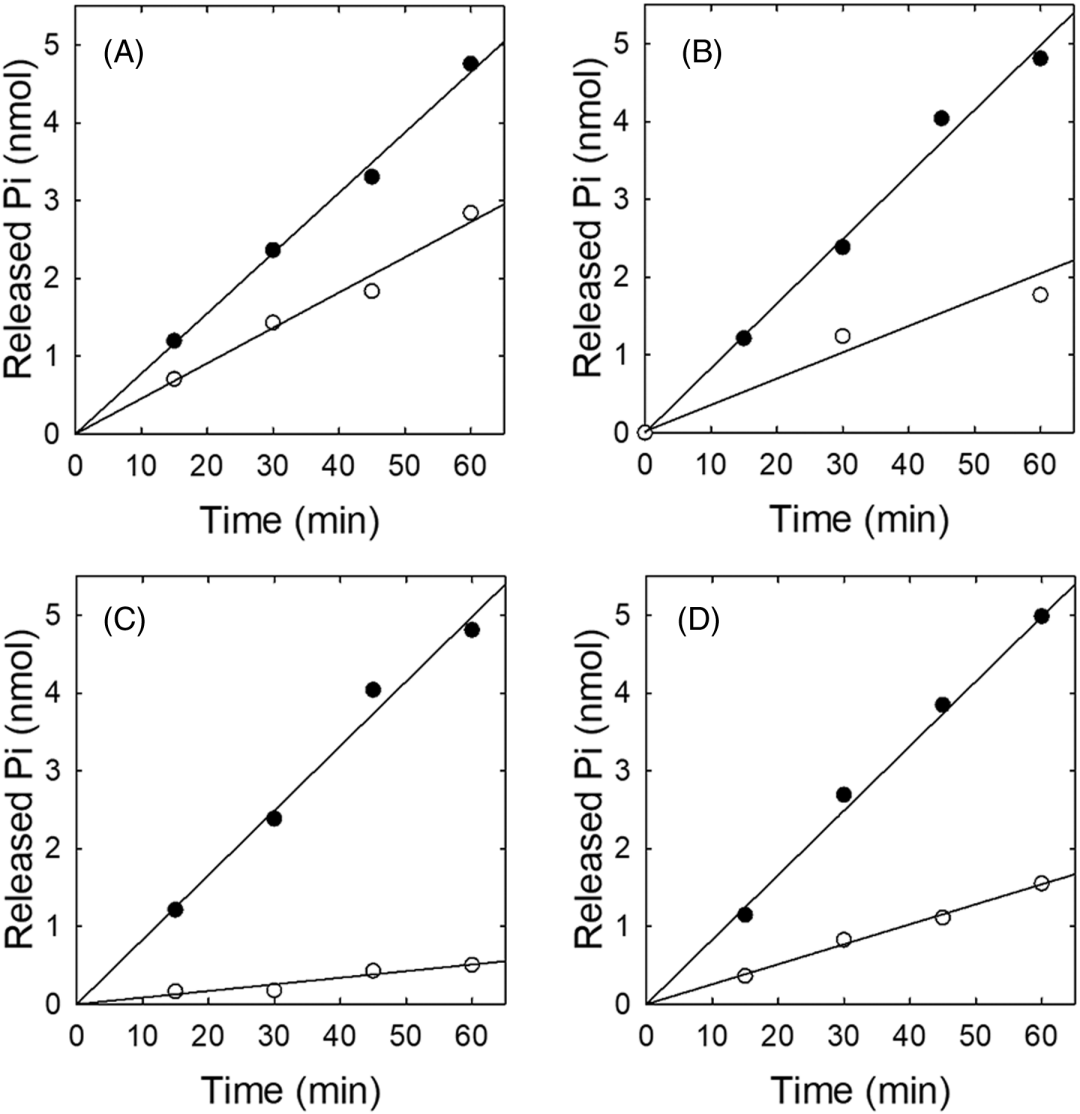
ATPase activity of BaCopA1. The rate of ATP hydrolysis was measured as described in Experimental Procedures. The composition of the reaction medium was that indicated in Experimental Procedures except the concentrations of one component specified in each case: (A, protein) sample with 5 μg purified enzyme (white circles) and 11 μg purified enzyme (black circles); (B, Cu^+^) sample without CuSO_4_ (white circles) and with 100 μM CuSO_4_ + 20 mM Cysteine (black circles); (C, Mg2^+^) sample without MgCl_2_ added (white circles) and with 2.0 mM MgCl_2_ (black circles); and (D, vanadate) sample without vanadate (black circles) and with 10 μM vanadate (white circles).

The effects of the typical modulators of the enzyme‐catalysed ATP hydrolysis are shown in Figure 5. The linear dependence of the release of Pi on the incubation time indicates that the enzyme does not inactivate in the window of time used, and the slope of each curve (relative to the amount of enzyme in the incubation media) gives the enzymatic activity (Table S3). For these assays, the composition of the incubation media was the same with the only exception of the concentration of the chemical species explored in each panel of the figure.

It can be observed that increasing the enzyme concentration produces a proportional increase in the rate of ATP hydrolysis (Figure 5A and Table S3). The ATPase activity of BaCopA1 increases when Cu(I) is included in the reaction medium, as shown in Figure 4, stating that this metal ion activates the enzyme (Figure 5B). Also, when Mg^2+^ is eliminated from the reaction mixture, BaCopA1‐mediated ATP hydrolysis is not significantly different from the non‐enzymatic rate (Figure 5C), showing that this cofactor is essential for function. Finally, an inhibitory effect of ATPase activity occurs with the addition of vanadate (Figure 5D), which is a specific inhibitor of P‐ATPase‐type enzymes (Clausen et al., 2016).

**FIGURE 6:**
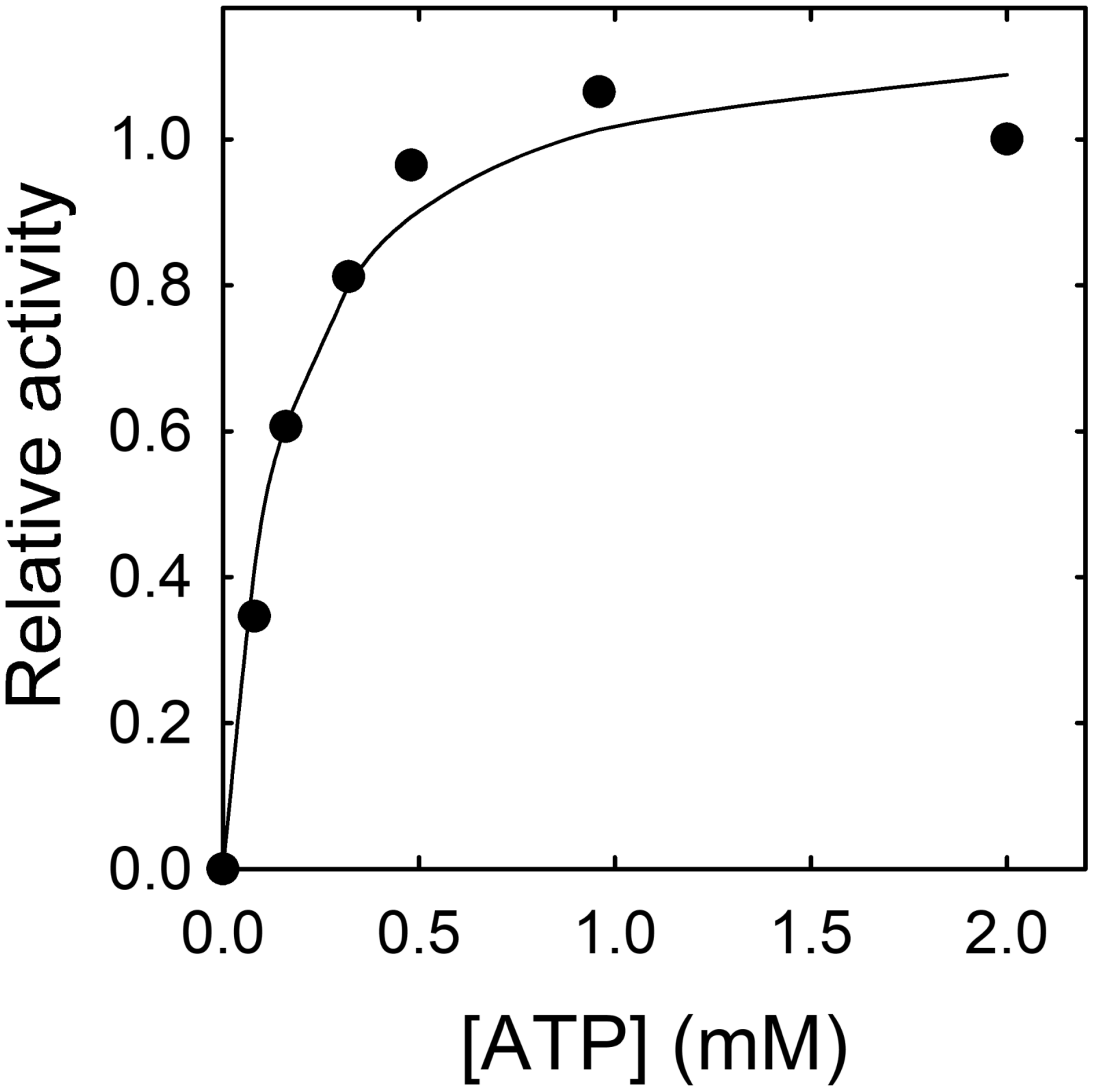
BaCopA1 ATPase activity dependence on ATP concentration. Purified BaCopA1 was incubated with increasing concentrations of ATP‐Mg^2+^ keeping constant the concentration of the other components of the reaction mixture (see Section 2). Free Mg^2+^ concentration was kept constant at 0.25 mM in all conditions. ATPase activity was obtained at each point as the initial rate of release of inorganic phosphate per milligram of enzyme and expressed as relative to the maximal enzyme activity (black circles). The line represents a hyperbolic function fitted to the experimental data. The best‐fit value of *K*_m,ATP_ was 0.15 ± 0.03 mM.

The substrate curve of BaCopA1 was obtained following the ATPase activity of the enzyme at different ATP concentrations (Figure 6). The best fit to the experimental data was obtained with a hyperbolic function of ATP concentration being *K*_m,ATP_ = 0.15 ± 0.03 mM. This value is close to that reported for AfCopA and LpCopA (Bredeston & González Flecha, 2016; Mandal et al., 2002; Placenti et al., 2022).

### BaCopA1 structural models

BaCopA1 shares a high sequence identity and similarity with LpCopA and AfCopA (Figure S7 and Table S2). All the residues of LpCopA identified as the TM copper pathway are conserved in BaCopA (Figure S4). The highest identity regions are found in the typical conserved motifs of P‐ATPases and in the TM helices TM4, TM5 and TM6, where the P_IB‐1_ subgroup of conserved residues and the TM Cu(I) site are located.

The experimentally determined structures of mesophile LpCopA (Andersson et al., 2014; Gourdon et al., 2011) and thermophile AfCopA (Salustros et al., 2022) allow us to generate three‐dimensional structure models for BaCopA1. The sequence of BaCopA1 was used as a template in a set of programs to obtain five models that represent different protein conformations (Table 3). Each model was evaluated for quality and aligned with the experimental structures of LpCopA and AfCopA, which are representative of the E2P, E2Pi, E1 and E1ATP conformations (Table 3, Figure S1). The root mean square deviation between each structural model and AfCopA‐E1 (7R0G), AfCopA‐E1‐ATP (7R0I), LpCopA‐E2P (4BBJ) and LpCopA‐E2Pi (3RFU) are shown in Table 3. Interestingly, the Alpha Fold 2 model has a lower RSMD value with AfCopA‐E1, meanwhile, the other models are more similar to LpCopA conformations. Then, we select the model structures obtained with Alpha Fold 2 (BaCopA1‐E1), MODELLER (BaCopA1‐E2P) and Tr‐Rosetta (BaCopA1‐E2Pi), as they have good structure quality parameters and can be shown as the conformers of BaCopA1 in different states of the reaction cycle.

**TABLE 3:** BaCopA1 structural models statistics.

Model	MP score	*Z*‐score	RMSD (Å)			
			AfCopA E1 (7R0G)	AfCopA E1‐ATP (7R0I)	LpCopA E2P (4BBJ)	LpCopA E2Pi (3RFU)
BaCopA‐Phyre2	3.05*	−1.52	11.494	11.682	1.734	0.538
**BaCopA‐AlphaFold2**	**2.09**	**2.34**	**1.829**	**2.363**	**9.321**	**11.531**
**BaCopA‐Modeller**	**2.39**	**1.49**	**9.515**	**9.537**	**0.347**	**1.655**
**BaCopA‐TrRosetta**	**2.73**	**−1.5**	**10.995**	**11.203**	**1.727**	**0.470**
BaCopA‐RosettaFold	3.27*	2.81	9.807	9.240	2.831	2.285

*Note*: The parameters MolProbity score (MP Score) and Ramachandran *Z*‐score (*Z*‐score) were established based on the quality assessment of BaCopA1 models. Root mean square deviation (RMSD) values were calculated after structure alignment between each model and each crystallographic structure. The best results are highlighted in bold. (*) in the MP score column, identify structures with MolProbity cutoff troubles.

Abbreviations: AfCopA, Cu(I) transport ATPases from *Archaeoglobus fulgidus*; LpCopA, Cu(I) transport ATPases from *Legionella pneumophila*.

**FIGURE 7:**
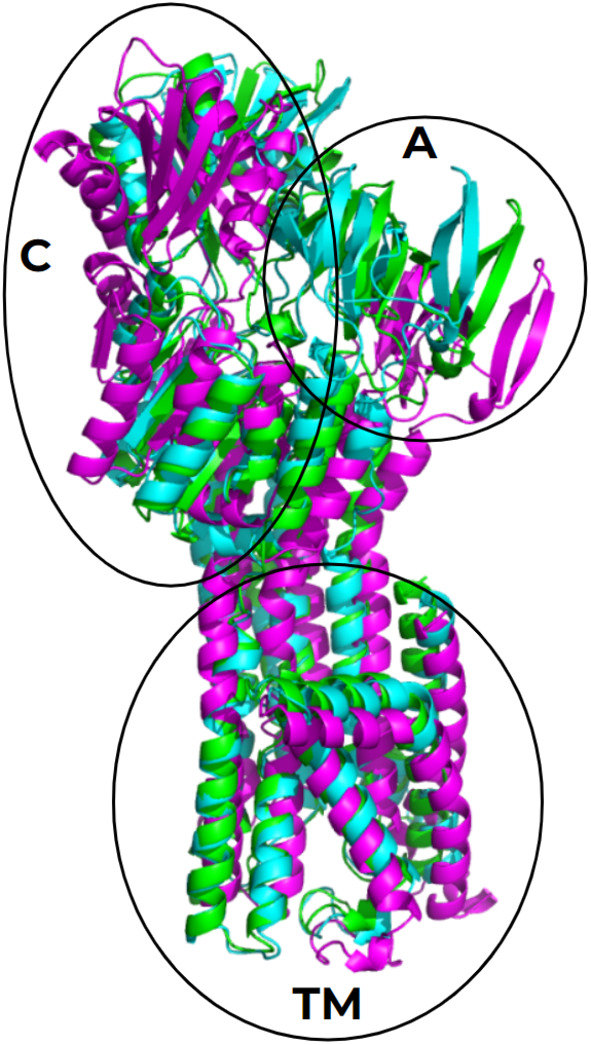
Aligned BaCopA1 structural models. BaCopA1 structural models that resemble different conformations were obtained as described in Experimental Procedures BaCopA1‐E1 (magenta), obtained with Alpha Fold 2, has the lowest root mean square deviation (RMSD) with AfCopA 7R0G, which was identified as the E1 conformation. BaCopA1‐E2P (green) was obtained with MODELLER and had the lowest RMSD with LpCopA 4BBJ, which was reported as the E2P conformation. BaCopA1‐E2Pi (cyan), obtained with Tr‐Rosetta, has the lowest RMSD with LpCopA 3RFU, which was reported as the E2Pi conformation. The actuator (A) catalytic (C) and transmembrane (TM) domains are highlighted in circles.

Figure 7 shows the structural alignment of the models corresponding to the conformations E1, E2P and E2Pi of BaCopA1. The main differences between these models are found in the position and orientation of the soluble actuator and catalytic domains, meanwhile, the TM domain showed fewer structural changes.

The structure of BaCopA1‐E1 (Panel A in Figure S8) shows the same inward facing‐conformation described for AfCopA (7R0G), with the TM Cu(I) binding site (C483 and C485) exposed to the intracellular side (Salustros et al., 2022). This arrangement could allow direct interaction between BaCopA1 and a copper chaperone for Cu(I) transfer. The catalytic and actuator domains of BaCopA1‐E1 are separated from each other and show the same orientation.

The AfCopA residues Y682, N683 (located at TM5) and M711, S715 (located at TM6), required for Cu(I) binding and enzyme activity (Mandal et al., 2004), are conserved in BaCopA1. The HP‐motif (H462, P463, I464), Gly‐rich region (G490, G492, G501) and phosphate binding site (T572, D574), involved in ATP binding into the catalytic domain of AfCopA (Tsuda & Toyoshima, 2009) are also conserved in BaCopA1. There is a Leu residue in the position of AfCopA I464, but this mutation is also present in LpCopA.

**FIGURE 8:**
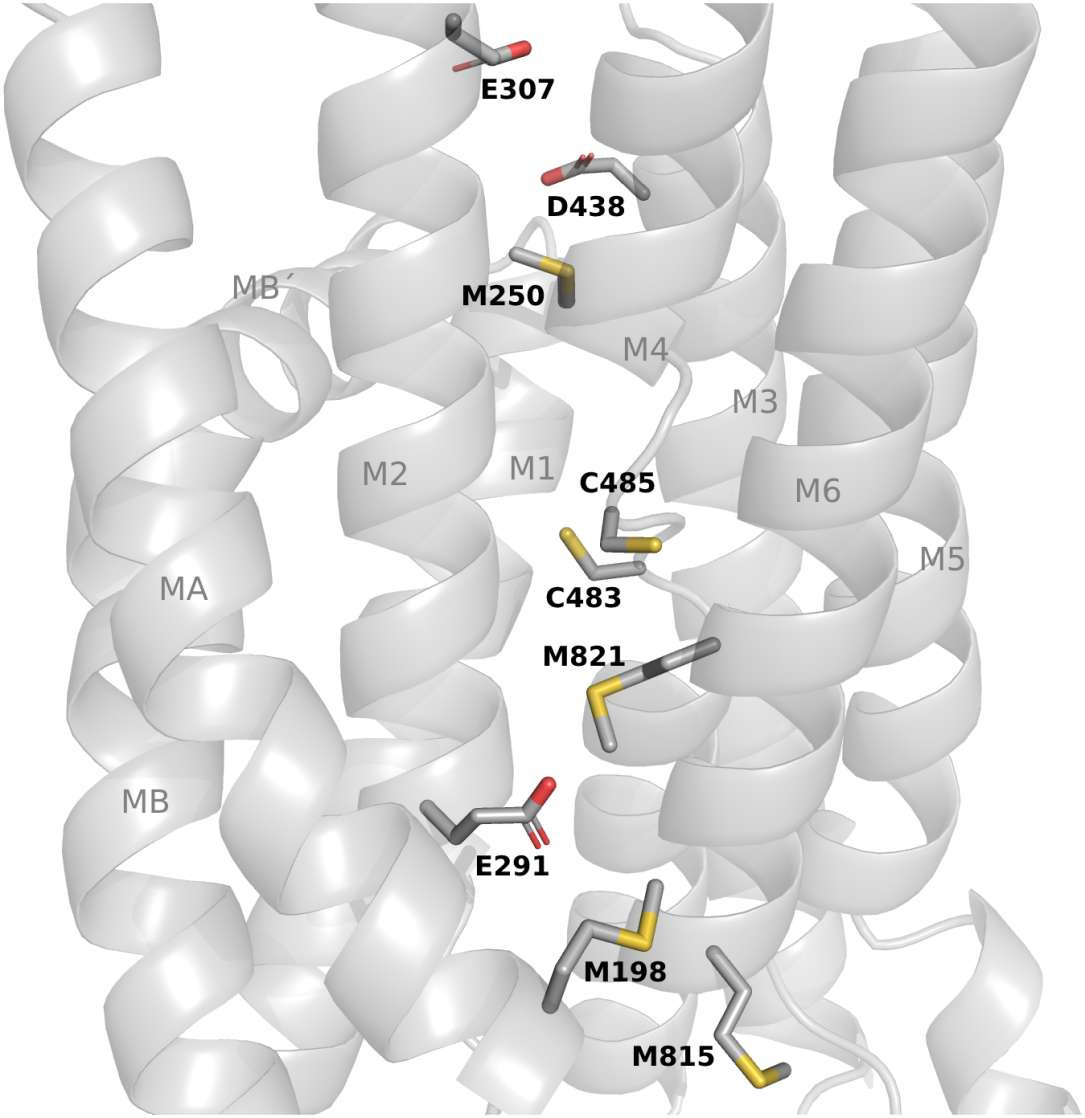
Putative transmembrane (TM) copper pathway of BaCopA1‐E2P model. The conserved residues that would be involved in the ion translocation process were identified according to the ones reported for LpCopA (4BBJ). The residues of the initial Cu(I) coordination site (M250, E307, D438), the high affinity TM site (C483, C485) and exit site (M198, E291, M815, M821) are shown as sticks. TM helices are labelled according to the reference names used for P_IB_‐ATPases (TMA–TMB, TM1–TM6).

Following Anderson et al. proposal for LpCopA (4BBJ), we set out to identify the metal‐binding residues of BaCopA1‐E2P (Panel B in Figure S8) that would be involved in the ion translocation process (Andersson et al., 2014). The conserved residues M250, E307 and D438 (equivalent to M148, E205 and D337 in LpCopA) are the candidates for the initial Cu(I) coordination site (Figure 8). The putative cytoplasmic chaperone of the CopZ family (G2EDT7) could interact with BaCopA1 near this initial Cu(I) coordination site, giving up Cu(I) ions to be transported to the periplasmic space. Indeed, it was demonstrated that mutation of these conserved residues in AfCopA impairs the activation driven by CopZ (Padilla‐Benavides et al., 2013).

The high‐affinity TM site would be conformed by the strongly conserved residues C483 and C485. The distance between the initial coordination site and the TM site (C483 and M250) is 8.9 Å, similar to the value reported for LpCopA (Andersson et al., 2014). After the conformational rearrangement produced in the reaction cycle, the ligand would move through M821 (equivalent to M717 in LpCopA) to the exit site conformed by M198, E291 and M815 (equivalent to M100, E189 and M711 in LpCopA).

The BaCopA‐E2Pi model (Panel C in Figure S8) shows that the TM domain remained almost unchanged in the shift from BaCopA‐E2P, meanwhile, the cytoplasmic domains moved closer to each other.

The conservation of essential residues and the structural similarity observed between BaCopA1 models with AfCopA and LpCopA strongly suggest that these proteins share the same reaction mechanism for copper ions transport.

## CONCLUDING REMARKS

Copper homeostasis is well described in model mesophilic bacteria, where several mechanisms related to uptake, transport and elimination are known (Giachino & Waldron, 2020; Novoa‐Aponte & Argüello, 2022). The sequencing of the genomic DNA of *B. argentinensis* allowed us to explore the genetic and functional aspects of copper resistance in cold‐adapted micro‐organisms (Andrade et al., 2001; Bercovich et al., 2008). As expected, a wide variety of proteins that could be related to copper regulation were found in the *B. argentinensis* genome: the TonB‐dependent Transporter for Cu(I) uptake, the periplasm Multicopper Oxidase for copper detoxification, periplasmic plastocyanin‐like copper chaperones for periplasm storage and transport, periplasmic chaperones for cuproprotein biogenesis, the intracellular chaperone CopZ for Cu(I) delivery, the CcoG like protein for Cu(II) reduction, the intracellular transcriptional factors ArsR‐SmtB for regulation, RND transporters for copper ions elimination and P_IB‐1_‐ATPases for Cu(I) active transport.

These peculiar proteins suggest that this bacterium (isolated from a copper‐rich marine environment in Antarctica) developed specific defence mechanisms, which might be related to cold adaptation. The presence of a large repertory of proteins that could fulfil roles in sensing, intracellular mobilisation and elimination of copper ions suggests a complex copper regulation system in *B. argentinensis*. Remarkably, this bacterium possesses a significant quantity of proteins involved in maintaining copper balance in the periplasmic region (Table S1). This particular compartment could play a vital role in monitoring external copper ion levels and capturing these ions to prevent harm to the cell (Ishihara et al., 2023).

It is broadly recognised that P_IB‐1_‐ATPases are crucial for copper resistance (Andrei et al., 2020), so we have focused our analysis on these proteins. The three sequences found in *B. argentinensis* show high similarity and identity with P_IB‐1_‐ATPases belonging to mesophilic and hyperthermophilic organisms. Several authors probe that this protein family is conserved among all domains of life, suggesting it has an essential role (Recoulat Angelini et al., 2021 and references therein).

In most organisms, two genes encode for Cu(I)‐ATPases (CopA‐1 and CopA‐2), and they exhibit different ion efflux rates due to their involvement in different copper regulatory sites. The presence of three P_IB‐1_‐ATPases in the *B. argentinensis* genome suggests more than two points of regulation. Other bacteria also show this diversity, e.g. *Sinorhizobium meliloti* harbours five Cu(I)‐ATPase, some of which play roles in the nitrogen cycle (Patel et al., 2014). Since *B. argentinensis* was identified as a denitrifying bacterium (Lanzarotti et al., 2011), its numerous repertoire of these types of Cu(I) transporters probably fulfils multiple functions similar to those reported for *S. meliloti*.

To further investigate the possible function of *B. argentinensis* P_IB‐1_‐ATPases in copper resistance, we chose one of these proteins for cloning, recombinant expression and characterisation. BaCopA1 shares the typical topological pattern of P_IB‐1‐_ATPases, has high sequence similarity with LpCopA and AfCopA, and is located in a TU near several putative copper‐related genes. These features were the reason for choosing it for recombinant cloning, expression and purification. The biochemical characterisation of BaCopA1 ATPase activity shows that this enzyme behaves similarly to other P_IB‐1_‐ATPases, which are activated by Cu(I), need the cofactor Mg^+2^ for reaction, and show a hyperbolic dependence on ATP concentration. BaCopA1 activity increased with temperature in the range of 5–28°C, but Cu(I) activation is higher in cold conditions. This might be an adaptation for copper elimination at low temperatures, suggesting that BaCopA1 plays a crucial role in cellular regulatory mechanisms in *B. argentinensis*. Notably, to our knowledge, this is the first report of a psychrotolerant P_IB‐1_‐ATPase being successfully expressed and purified in a functional form.

The structural models of BaCopA1 show structural similarities with AfCopA (Salustros et al., 2022) and LpCopA (Andersson et al., 2014; Gourdon et al., 2011). Structural analysis of these models shows that the residues involved in the TM Cu(I) pathway are highly conserved in this sub‐family (Andersson et al., 2014), and some residues critical for ATP binding (Sazinsky et al., 2006) and Cu(I) transfer to chaperones (González‐Guerrero & Argüello, 2008) also show a high percentage of structural conservation with respect to LpCopA and AfCopA. These findings strongly suggest that BaCopA1 shares the same reaction mechanism for copper transport as both mesophilic and hyperthermophilic counterparts.

This novel data lays the groundwork for future investigations into the mechanisms governing copper regulation and transport in psychrotolerant organisms. Furthermore, these findings have potential implications for understanding microbial resistance to copper and exploring applications in environmental and biotechnological contexts.

## AUTHOR CONTRIBUTIONS

**Noelia I. Burgardt:** Conceptualization (equal); formal analysis (equal); funding acquisition (supporting); investigation (equal); methodology (equal); supervision (equal); writing – original draft (equal); writing – review and editing (equal). **Noelia A. Melian:** Formal analysis (equal); investigation (equal); methodology (equal); writing – review and editing (equal). **F. Luis González Flecha:** Conceptualization (equal); funding acquisition (lead); supervision (equal); validation (equal); writing – original draft (equal); writing – review and editing (equal).

## CONFLICT OF INTEREST STATEMENT

The authors declare no conflict of interest.

## Supporting information


**Data S1.** Supporting information.

## Data Availability

All data generated or analysed during this study are included in this article.
