# Supplementary material for: Copper resistance in the cold: Genome analysis and characterisation of a PIB‐1 ATPase in Bizionia argentinensis
Source: Environ Microbiol Rep. 2024 Jun 28;16(4):e13278. doi: 10.1111/1758-2229.13278 (PMC11213822; doi:10.1111/1758-2229.13278)
Supplement: Supplementary file 1 — Data S1. Supporting information. [file EMI4-16-e13278-s001.pdf]

Supplementary information

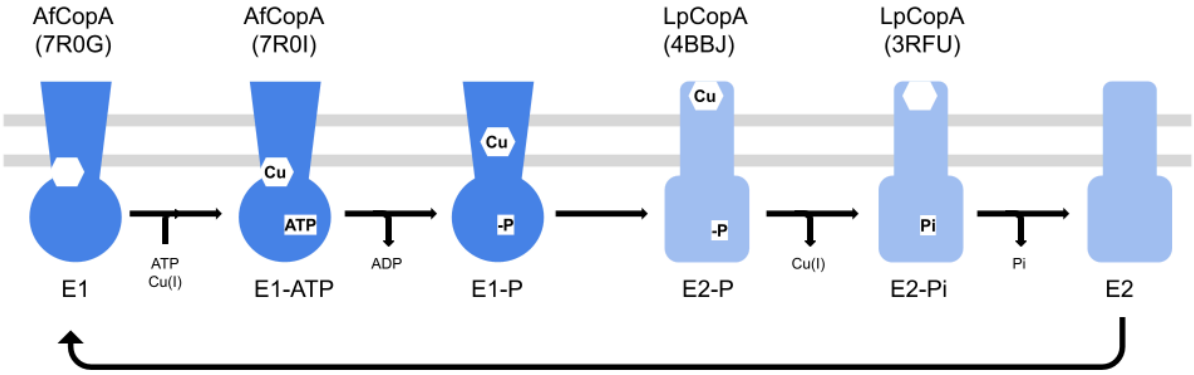

FIGURE S1. Schematic representation of the Albers-Post reaction cycle. P_{IB-1}-ATPases enzymatic mechanism is described by the Albers-Post model, which proposes two enzyme conformations denoted as E1 and E2 (Recoulat Angelini *et al*. 2021 and references therein). The cycle starts when the inward-facing E1 conformation binds Cu(I) and ATP, resulting in the E1-ATP form. Afterwards, the nucleotide is hydrolyzed, and the enzyme gets phosphorylated given the E1P conformation. Then, a conformational change forms the outward-facing E2P state and the Cu(I) is released, leading to the dephosphorylated form (E2Pi). Finally, the phosphate anion is released and the pump returns to the E1 conformation closing the cycle. The corresponding conformations of AfCopA and LpCopA experimentally resolved structures are indicated in the top of each state of the reaction cycle.

```
P36649    AAKGQYER--WVIS----------------------GVGDMMLHPFHIHGTQFRILSENG   459
G2EDV6    NLTGNMQRYIWSMNGVPLSEADNIKIKGGEVTRMTFNNLTMMHHPMHLHGHFFRVINKNG   559
Q47452    HLTGHMEKFAWSFNGIKFSDAAPVLLKYGERLRITLINDTMMTHPIHLHGMWSDLEDENG   554
            .*: ::  * :.                          ** **:*:**    : .:**

P36649    KPPAAHRAGWKDTVKVEGNVSEVLVKF-NHDAPKEHAYMAHCHLLEHEDTGMMLGFTV--   516
G2EDV6    DYSPLK-----HTVNVPPMQ-EVTIEFSGNNGDEYGDWFFHCHILYHMMSGMARVVSYDT   613
Q47452    NFMVRK-----HTIDVPPGT-KRSYRV---TADALGRWAYHCHLLYHMEMGMFREVRVEE   605
          .    :     .*:.*     :   ..    .     :  ***:* *   **   .
```

FIGURE S2. Multiple alignment section between *E. coli* CueO (P36649), *E.coli* PcoA (Q47452) and *B. argentinensis* Multicopper oxidase (G2EDV6). Conserved residues at the copper binding site are highlighted in blue (Roberts. *et al.* 2002 ,Bhamidimarri *et al*. 2021) and important residues (methionines) at the entrance to the copper site are highlighted in green.

```
G2ED03    -MKK-HLLVLAVLALIFSVSCENANKKENTDSDPLDVEKTDTELKLDIHTSQTSLDWKGT    58
A7ZHT3    MVKKAIVTAMAVISLFTLMGCNNRAEVDTLS--PA--------------QAAELKPMPQS    44
           :**  : .:**::*:  :.*:*  : :. .  *                :        :

G2ED03    YVGTLPCADCEGIKTTIRLNEDLTYDAVMEYLGKEENSVGSKGYYKWSDDGLNIILSDDT   118
A7ZHT3    WRGVLPCADCEGIETSLFLEKDGTWVMNERYLGAREEPSSFASYGTWARTADKLVLTDSK   104
          : *.*********:*:: *::* *:    .*** .*:  .  .* .*:  . :::*:*..
```

FIGURE S3. N-terminal portion of the alignment between *E. coli* CutF (A7ZHT3) and the putative *B. argentinensis* chaperone (G2ED03). Conservation of the copper-binding site motif (CXXC) is shown in blue, and the periplasmic peptide signal in orange (Öztürk *et al*. 2021). Complete alignment indicates 35% identity and 52% sequence similarity.

TM4

```
                                     CPC
G2EDW3      VYALVNAIAVLIIACPCALGLAT
G2EGV6      VYALVNAIAVLIIACPCALGLAT
G2EC62      SKAMNVFTAVLIIVCPCAIALSA
G2EHA4      NESLYRAITVLVAASPCALAIST
```

TM5

```
                          YN(X)4P
G2EDW3      NLFFALIYNTLGVPIAAGVLFPI
G2EGV6      NLFFALIYNTLGVPIAAGVLFPI
G2EC62      SFVLSFFYNIIGLYFAVTGQLEP
G2EHA4      NIFISLGVVAILVPVTILGLTNI
```

TM6

```
                              M(X)2SS
G2EDW3      ILLSPMIAALAMSFSSVSVIAN
G2EGV6      LLLSPMIAALAMSFSSVSVIAN
G2EC62      GQLEPVIAAILMPLSSISIVAF
G2EHA4      GLTNIGLAVLFHEGSTIVVVLN
```

FIGURE S4. TM helix sequence analysis of P_{IB}-ATPases from *B. argentinensis*. The alignment of helices TM4, TM5 and TM6 of the four P_{IB}-ATPases (G2EDW3, G2EGV6, G2EC62, G2EHA4) from *B. argentinensis* is shown. The ion specificity of P_{IB}-ATPases can be predicted from the conserved motif present in each sequence (Argüello 2003). The conserved motif of the IB-1 subtype is highlighted in red bold and the subtype IB-4 is highlighted in black bold. As a result, the proteins can be classified as follow: G2EDW3, G2EGV6, G2EC62 are P_{IB-1}-ATPases (BaCopA1, BaCopA2 and BaCopA3) and G2EHA4 is a P_{IB-4}-ATPase (BaCzcP).

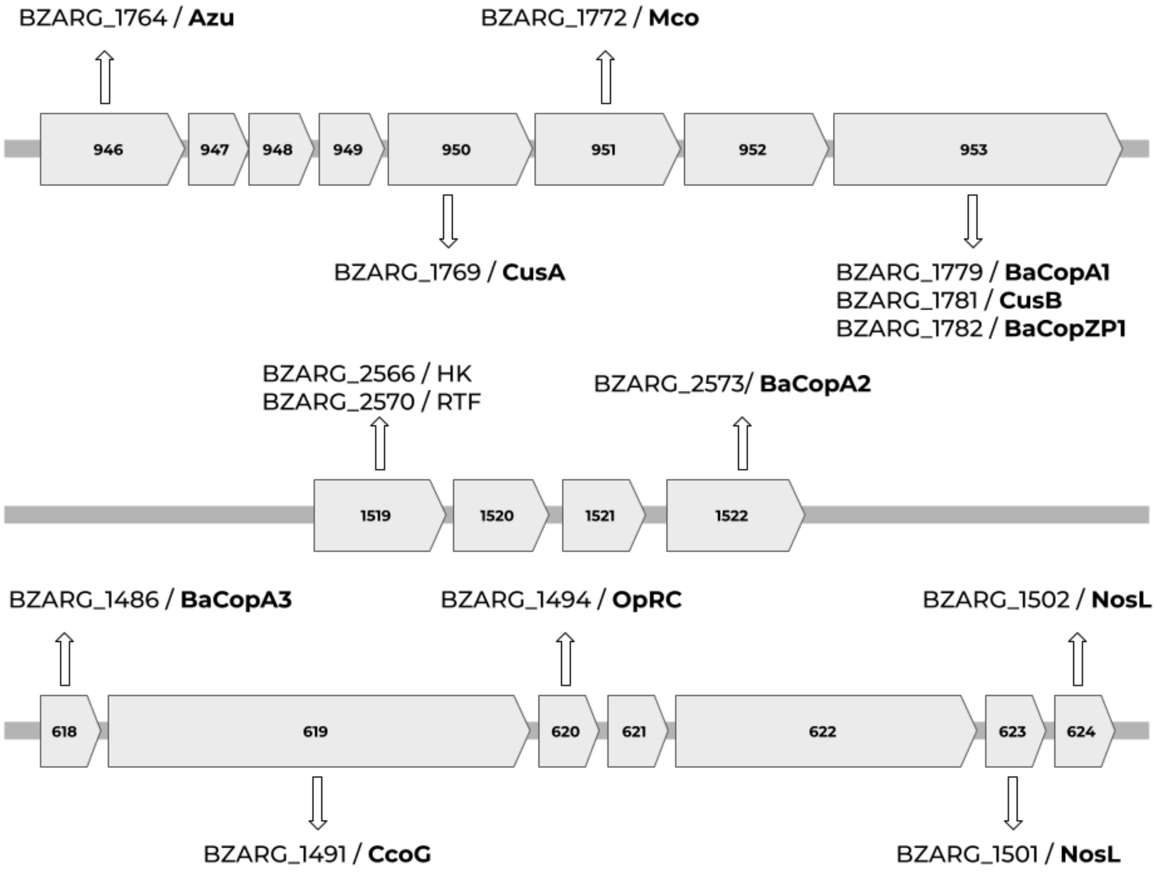

FIGURE S5. Schematic representation of transcriptional units found in *B. argentinensis* genome containing open reading frames of copper homeostasis related proteins. Potential P_{IB-1}-ATPase genes are located either within the same transcriptional unit (TU) or in close proximity to other genes associated with copper homeostasis. Specifically, BaCopA1 is part of TU 953, along with the potential periplasmic chaperone (BaCopZP1) and subunit B of the RND transporter (CusB). Upstream TU 953 there are TUs coding for azurin (Azu), a multicopper oxidase (Mco), and subunit A of the RND transporter (cusA). BaCopA2 is located in TU 1522 far away from other genes associated with copper resistance, but close to TU 1519 where a two component regulation system is coded (HK: Histidine kinase, RTF: Regulator transcription factor). The gene encoding BaCopA3 is in TU 618, followed downstream by other TU with genes encoding for the cytochrome oxidase c accessory protein (CcoG), a copper entry transporter (OprC), and an auxiliary chaperone (NosL).

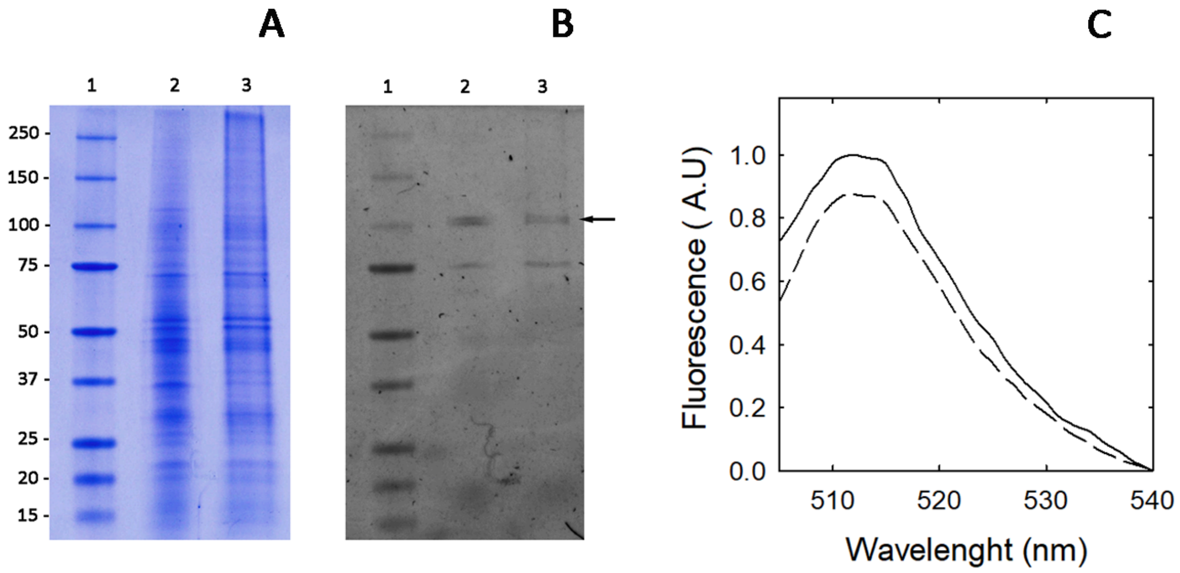

FIGURE S6. BaCopA1 solubilization assay. Recombinant BaCopA1 was expressed in *S. cerevisiae* and the membrane fraction was obtained following the protocol described in Experimental Procedures. SDS-PAGE analysis of total membranes (lane 2) and detergent-solubilized membranes (lane 3) is shown in Panel A. The pre-stained molecular weight markers are shown in lane 1, and the corresponding molecular weights are indicated in kDa. Panel B shows the image of the same gel before staining with Coomassie blue and trans illuminated with UV-vis light. A band corresponding to the molecular weight of BaCopA1 (plus GFP) is observed both in total membranes and in detergent-solubilized membranes. Panel C shows the fluorescence spectrum of a sample of 20 μl of total membranes resuspended up to 200 μl in YSB 1X (solid line) and the same volume of membranes solubilized with DDM and resuspended up to 200 μl in YSB 1X (dashed line) upon excitation at 425 nm. These results indicate that BaCopA1 is properly localized in the membrane fraction and that the selected detergent is able to solubilize nearly 80% of BaCopA1 from the membranes.

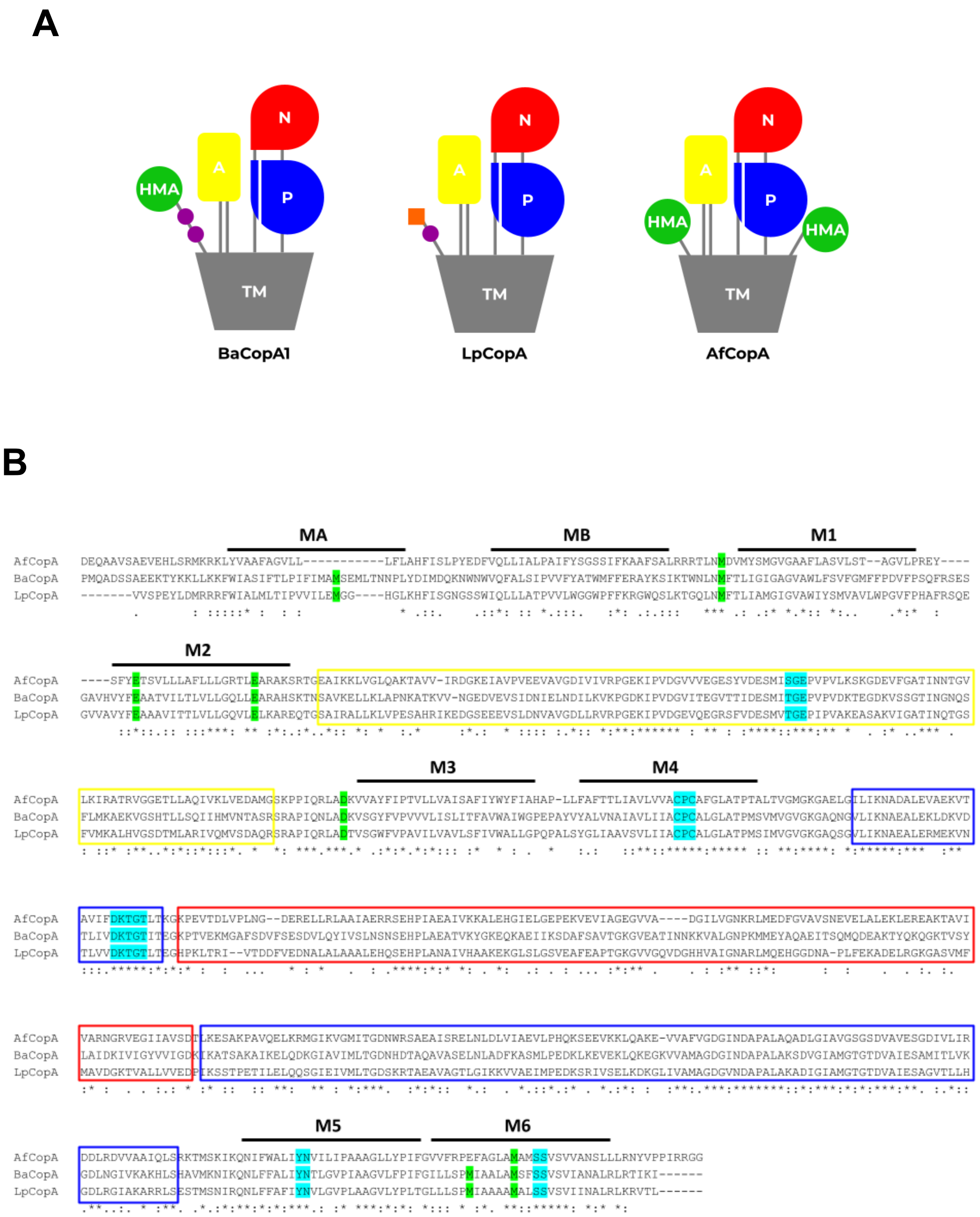

FIGURE S7. Comparison between CopA homologues. (A) Schematic comparison among Cu(I)-transporting ATPases domains from *L. pneumophila* (LpCopA), *A. fulgidus* (AfCopA) and *B. argentinensis* (BaCopA1). B) Sequence alignment of these proteins without the soluble MBD. It can be observed that these proteins share a highly similar common core of domains: the TM domain (black lines),the actuator domain (A, boxed in yellow) the phosphorilation domain (P, boxed in blue) , the nucleotide binding domain (N, boxed in red) and a slightly variable set of metal binding domains Residues involved in the LpCopA transmembrane copper pathway are highlighted in green and residues conserved in all the P-ATPase family are highlighted in cyan.

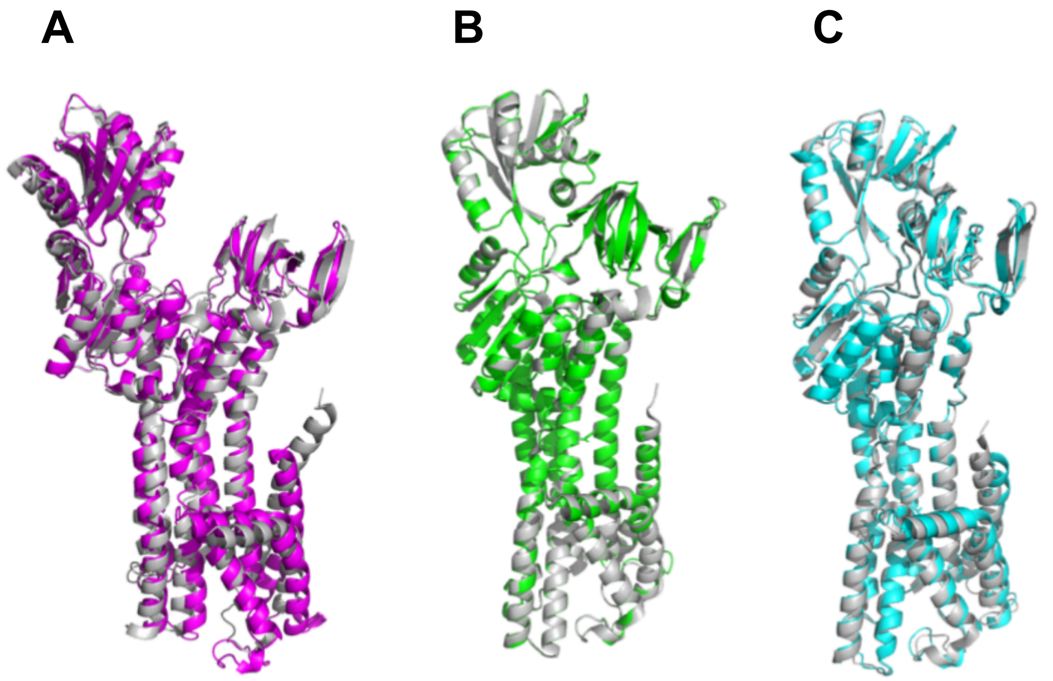

FIGURE S8. Structural alignments between models and experimental structures. Panel A shows the model for BaCopA1-E1 (magenta) aligned with the experimental structure of AfCopA-E1 (7R0G, gray). Panel B includes the alignment between the model for BaCopA1-E2P (green) and the experimental structure of LpCopA-E2P (4BBJ, gray). Panel C shows the aligned structures of the model BaCopA1-E2Pi (cyan) and those experimentally determined for LpCopA-E2Pi (3RFU, gray).

TABLE S1. Reference proteins used as seed for *B. argentinensis* genome Blast-P search and the results obtained with their corresponding uniprot entry.

UniProt	Organism	Reference Protein	Function	Blast-P result
**P72121**	***Pseudomonas aeruginosa***	**TonB-dependent Transporter OprC**	**Outer membrane uptake**	**Positive (G2EC70)**
P02931	*Escherichia coli*	Copper specific porin OmpF	Outer membrane uptake	Negative
P06996	*Escherichia coli*	Copper specific porin OmpC	Outer membrane uptake	Negative
P0AB40	*Escherichia coli*	Multiple stress resistance BhsA	Outer membrane uptake	Negative
A0A542V2Q7	*Pseudomonas sp.*	Methanobactin biosynthesis MbnT	Outer membrane uptake	Negative
**Q47452**	***Escherichia coli***	**Copper resistance protein A PcoA**	**Periplasm resistance**	**Positive (G2EDV6)**
**P36649**	***Escherichia coli***	**Multicopper oxidase CueO**	**Periplasm resistance**	**Positive (G2EDV6)**
Q8ZL99	*Salmonella typhimurium*	Cu-binding protein CueP	Periplasm resistance	Negative
Q5F665	*Neisseria gonorrhoeae*	Copper storage protein Csp1	Periplasm resistance	Negative
P77214	*Escherichia coli*	Copper chaperone CusF	Periplasm resistance	Negative
**W8FLH9**	***Rubrivivax gelatinosus***	**Plastocyanin-like protein CopI**	**Periplasm resistance**	**Positive (G2EDU8 and G2EAM2)**
P12376	*Pseudomonas syringae*	Copper resistance protein CopC	Periplasm resistance	Negative
**Q52720**	***Rhodobacter capsulatus***	**ScoI-like chaperones SenC / BsSco**	**Periplasm biogenesis**	**Positive (G2ECM7 and G2EFU0)**
**Q9HYK8**	***Pseudomonas aeruginosa***	**NosL chaperones PaNosL / SdNosL**	**Periplasm biogenesis**	**Positive (G2EC77)**
**Q12M31**	***Shewanella denitrificans***	**NosL chaperones PaNosL / SdNosL**	**Periplasm biogenesis**	**Positive (G2EC77)**
Q5SGY7	*Thermus thermophilus*	Chaperone PCuAC-like PccA	Periplasm biogenesis	Negative
Q92RG6	*Rhizobium*	Chaperone Cox11	Periplasm	Negative

	meliloti		biogenesis	
Q9HV84	*Pseudomonas aeruginosa*	Copper binding CopG	Periplasm biogenesis	Negative
**A7ZHT3**	***Escherichia coli***	**Lipoprotein CutF / NlpE**	**Periplasm biogenesis**	**Positive (G2ED03)**
Q8ZL99	*Salmonella typhimurium*	Periplasmic protein CueP	Periplasm biogenesis	Negative
D5AKT2	*Rhodobacter capsulatus*	MFS-1 CcoA-like transporter	Inner membrane import	Negative
**O30731**	***Rhodobacter capsulatus***	**Copper-reducing enzyme CcoG**	**Intracellular resistance**	**Positive (G2EC67)**
**Q9HY89**	***Pseudomonas aeruginosa***	**Chaperone PaCopZ1**	**Intracellular resistance**	**Positive (G2EDT7)**
**O32221**	***Bacillus subtilis***	**Copper chaperone BsCopZ**	**Periplasm resistance**	**Positive (G2EDG9, G2EHB8, G2EDW6)**
A0A0H2ZPL4	*Streptococcus pneumoniae*	Chaperone CupA	Intracellular resistance	Negative
P30331	*Synechococcus elongatus*	Metallothionein - SmtA	Intracellular resistance	Negative
**O07571**	***Bacillus subtilis***	**Copper storage protein YhjQ-Csp3**	**Intracellular resistance**	**Positive (A0A4U8UH91)**
A0A4R8HID5	*Methylosinus sp.*	Methanobactin biosynthesis MbnM	Inner membrane export	Negative
**P77211**	***Escherichia coli***	**Cation efflux system protein CusC**	**Export**	**Positive (G2EF75)**
**P38054**	***Escherichia coli***	**Cation efflux system protein CusA**	**Export**	**Positive (G2EHA6, G2EDV3, G2EF74 and G2EHL7)**
**P77239**	***Escherichia coli***	**Cation efflux system protein CusB**	**Export**	**Positive (G2EDW5)**
**Q59385**	***Escherichia coli***	**Copper-exporting P-type ATPase**	**Export**	**Positive (G2EDW3, G2EGV6, G2EHA4 and G2EC62)**
**P0A9G4**	***Escherichia coli***	**Transcriptional regulator CueR**	**Cytoplasm regulation**	**Positive (G2EA47 and G2E8Y3)**
**Q47839**	***Enterococcus hirae***	**Transcriptional repressor CopY**	**Cytoplasm regulation**	**Positive (G2EAK1, G2ED45 and G2EAI2)**

**Q76L30**	***Oscillatoria brevis***	**Transcriptional repressor BxmR**	**Cytoplasm regulation**	**Positive (G2EAK5 and G2E9K0)**
P9WP49	*Mycobacterium tuberculosis*	Transcriptional repressor CsoR	Cytoplasm regulation	Negative
**P0ACZ8**	***Escherichia coli***	**Transcriptional regulatory protein CusR**	**Periplasm regulation**	**Positive (G2EGV3, G2EGQ4 and G2EHH1)**
**P77485**	***Escherichia coli***	**Sensor histidine kinase CusS**	**Periplasm regulation**	**Positive (G2EHH2 and G2EGQ3)**
**Q9I034**	***Pseudomonas aeruginosa***	**Two-component response regulator CopR**	**Periplasm regulation**	**Positive (G2EGV3, G2EGQ4, G2EHH1 and G2EGE5)**
**Q02541**	***Pseudomonas syringae***	**Sensor protein CopS**	**Periplasm regulation**	**Positive (G2EHH2, G2EBH0 and G2EGX1)**
**Q9I033**	***Pseudomonas aeruginosa***	**Two-component response regulator CopS**	**Periplasm regulation**	**Positive (G2EHH2, G2EGQ3 and G2EBH0**
Q47453	*Escherichia coli*	Copper resistance protein B PcoB	Outer membrane uptake	Negative
Q47454	*Escherichia coli*	Copper resistance protein C PcoC	Periplasm resistance	Negative
Q47455	*Escherichia coli*	Copper resistance protein D PcoD	Inner membrane uptake	Negative
Q47459	*Escherichia coli*	Probable copper-binding protein PcoE	Periplasm resistance	Negative
**Q47456**	***Escherichia coli***	**Transcriptional regulatory protein PcoR**	**Periplasm regulation**	**Positive (G2EGV3, G2EGQ4 and G2EHH1)**
**Q47457**	***Escherichia coli***	**Probable sensor protein PcoS**	**Periplasm regulation**	**Positive (G2EHH2, G2EGQ3 and G2EBH0)**

Note: The sequences of bacterial proteins involved in copper ions homeostasis were used as seed for Blast-P search of similar proteins on the *B. argentinensis* genome using NCBI server. This table shows all, the positive and negative results of the blast search. When positive results were obtained, further analysis was done to confirm the presence of essential motifs, signal peptides, membrane regions and others. After that, the proteins of *B. argentinensis* possibly related to copper management were selected and described in the manuscript.

TABLE S2. Identity and similarity of the aminoacid sequences of BaCopA1, BaCopA2 and BaCopA3 with the sequences of LpCopA and AfCopA.

UniProt	Name	LpCopA		AfCopA	
		Identity %	Similar %	Identity %	Similar %
G2EDW3	BaCopA1	51.8	80.8	40.3	73.9
G2EGV6	BaCopA2	52.8	82.2	39.8	75.1
G2EC62	BaCopA3	25.2	59.3	24.5	58.3

Note: Each pair of sequences were analyzed with Lalign Pairwise Sequence Alignment (PSA) server (Huang and Miller 1991) in order to find the identity and similarity percentage (%). Each CopA from *B. argentinensis* (their Uniprot code and name are shown) was compared to LpCopA (Uniprot code Q5ZWR1) and AfCopA (Uniprot code O29777).

TABLE S3. BaCopA1 ATPase activity values corresponding to the conditions depicted in the panels of Figure 5.

	**Conditions**	**Activity** (nmol h^{-1} μg^{-1})
**PANEL A**	5 µg purified enzyme	0.5452 ± 0.021
	11 µg purified enzyme	0.4228 ± 0.008
**PANEL B**	without copper	0.1845 ± 0.079
	100 µM $CuSO_4$	0.4528 ± 0.079
**PANEL C**	without Mg^{+2}	0.0464 ± 0.004
	2.0 mM Mg^{+2}	0.4528 ± 0.014
**PANEL D**	without vanadate	0.4605 ± 0.009
	10 µM vanadate	0.1405 ± 0.003

Note: The values are obtained from the slope of each curve and normalized by protein mass.

REFERENCES

Argüello JM (2003) Identification of ion-selectivity determinants in heavy-metal transport P1B-type ATPases. J Membr Biol 195:93-108. Available from: https://doi.org/10.1007/s00232-003-2048-2

Bhamidimarri SP, Young TR, Shanmugam M, Soderholm S, Baslé A, Bumann D, van den Berg B (2021) Acquisition of ionic copper by the bacterial outer membrane protein OprC through a novel binding site. PLoS Biol 19:e3001446. Available from: https://doi.org/10.1371/journal.pbio.3001446

Huang, X, Miller W (1991) A time-efficient, linear-space local similarity algorithm. Adv Appl Math 12: 337–357. Available from: https://doi.org/10.1016/0196-8858(91)90017-D

Öztürk Y, Blaby-Haas CE, Daum N, Andrei A, Rauch J, Daldal F, Koch HG.(2021) Maturation of Rhodobacter capsulatus Multicopper Oxidase CutO Depends on the CopA Copper Efflux Pathway and Requires the cutF Product. Front Microbiol.12:720644. Available from: https://doi.org/10.3389/fmicb.2021.720644

Recoulat Angelini AA, Placenti MA, Melian NA, Sabeckis ML, Burgardt NI, González Lebrero RM, Roman EA, González Flecha FL (2021) Cu(I)-Transport ATPases. Molecular architecture, catalysis and adaptation to extreme environments. Ad Med Biol 180:65-130.

Roberts SA, Weichsel A, Grass G, Thakali K, Hazzard JT, Tollin G, Rensing C, Montfort WR.(2002) Crystal structure and electron transfer kinetics of CueO, a multicopper oxidase required for copper homeostasis in *Escherichia coli*. Proc Natl Acad Sci U S A 99:2766-71. Available from: https://doi.org/10.1073/pnas.052710499
